# Childhood intelligence in relation to major causes of death in 68 year follow-up: prospective population study

**DOI:** 10.1136/bmj.j2708

**Published:** 2017-06-28

**Authors:** Catherine M Calvin, G David Batty, Geoff Der, Caroline E Brett, Adele Taylor, Alison Pattie, Iva Čukić, Ian J Deary

**Affiliations:** 1 Department of Psychology, University of Edinburgh, Edinburgh, EH8 9JZ, UK; 2 Centre for Cognitive Ageing and Cognitive Epidemiology (CCACE), Department of Psychology, University of Edinburgh, Edinburgh, EH8 9JZ, UK; 3 Department of Psychiatry, University of Oxford, Warneford Hospital, Oxford OX3 7JX, UK; 4 Department of Epidemiology and Public Health, University College London,; London, WC1E 6BT, UK; 5 MRC/CSO Social and Public Health Sciences Unit, University of Glasgow, Glasgow G2 3QB; 6 Natural Sciences and Psychology, Liverpool John Moores University, Tom Reilly Building, Liverpool, L3 3AF, UK

## Abstract

**Objectives** To examine the association between intelligence measured in childhood and leading causes of death in men and women over the life course.

**Design** Prospective cohort study based on a whole population of participants born in Scotland in 1936 and linked to mortality data across 68 years of follow-up.

**Setting** Scotland.

**Participants** 33 536 men and 32 229 women who were participants in the Scottish Mental Survey of 1947 (SMS1947) and who could be linked to cause of death data up to December 2015.

**Main outcome measures** Cause specific mortality, including from coronary heart disease, stroke, specific cancer types, respiratory disease, digestive disease, external causes, and dementia.

**Results** Childhood intelligence was inversely associated with all major causes of death. The age and sex adjusted hazard ratios (and 95% confidence intervals) per 1 SD (about 15 points) advantage in intelligence test score were strongest for respiratory disease (0.72, 0.70 to 0.74), coronary heart disease (0.75, 0.73 to 0.77), and stroke (0.76, 0.73 to 0.79). Other notable associations (all P<0.001) were observed for deaths from injury (0.81, 0.75 to 0.86), smoking related cancers (0.82, 0.80 to 0.84), digestive disease (0.82, 0.79 to 0.86), and dementia (0.84, 0.78 to 0.90). Weak associations were apparent for suicide (0.87, 0.74 to 1.02) and deaths from cancer not related to smoking (0.96, 0.93 to 1.00), and their confidence intervals included unity. There was a suggestion that childhood intelligence was somewhat more strongly related to coronary heart disease, smoking related cancers, respiratory disease, and dementia in women than men (P value for interactions <0.001, 0.02, <0.001, and 0.02, respectively).****Childhood intelligence was related to selected cancer presentations, including lung (0.75, 0.72 to 0.77), stomach (0.77, 0.69 to 0.85), bladder (0.81, 0.71 to 0.91), oesophageal (0.85, 0.78 to 0.94), liver (0.85, 0.74 to 0.97), colorectal (0.89, 0.83 to 0.95), and haematopoietic (0.91, 0.83 to 0.98). Sensitivity analyses on a representative subsample of the cohort observed only small attenuation of the estimated effect of intelligence (by 10-26%) after adjustment for potential confounders, including three indicators of childhood socioeconomic status. In a replication sample from Scotland, in a similar birth year cohort and follow-up period, smoking and adult socioeconomic status partially attenuated (by 16-58%) the association of intelligence with outcome rates.

**Conclusions** In a whole national population year of birth cohort followed over the life course from age 11 to age 79, higher scores on a well validated childhood intelligence test were associated with lower risk of mortality ascribed to coronary heart disease and stroke, cancers related to smoking (particularly lung and stomach), respiratory diseases, digestive diseases, injury, and dementia.

## Introduction

Findings from prospective cohort studies based on populations from Australia, Sweden, Denmark, the US, and the UK indicate that higher cognitive ability (intelligence) measured with standard tests in childhood or early adulthood is related to a lower risk of total mortality by mid to late adulthood.^1^ The association is evident in men and women^1^ ^2^; is incremental across the full range of ability scores^2^ ^3^; and does not seem to be confounded by socioeconomic status of origin or perinatal factors.^1^ ^4^ ^5^ Whereas similar gradients are also apparent for selected causes of death, such as cardiovascular disease,^3^ ^6^ ^7^ ^8^ ^9^ ^10^ suicide,^7^ ^10^ ^11^ ^12^ ^13^ ^14^ ^15^ and injuries,^7^ ^16^ ^17^ the association with other leading causes remains inconclusive or little tested. Mortality surveillance for the entire population of one country born in 1936 who had an assessment of childhood intelligence provides the valuable opportunity to examine little tested associations between intelligence and mortality and consider specificity by exploring the strengths of these associations according to leading causes of death, in men and women, and over almost the entire life course.

Several hypotheses have been proposed to explain associations between intelligence and later risk of mortality.^18^ The suggested causal mechanisms put forward, in which cognitive ability is the exposure and disease or death the outcome, include mediation by adverse or protective health behaviours in adulthood (such as smoking, physical activity), disease management and health literacy, and adult socioeconomic status (which could, for example, indicate occupational hazards).^18^ Recent evidence of a genetic contribution to the association between general cognitive ability and longevity,^19^ however, might support a system integrity theory that posits a “latent trait of optimal bodily functioning” proximally indicated by both cognitive test performance and disease biomarkers.^20^ None of these possibilities are mutually exclusive. Whereas cognitive epidemiology^18^ makes a unique contribution to improved understanding of health inequalities in populations, by its successful application of a well validated behavioural trait that performs independently of social gradients in its association with health indices,^21^ ^22^ there remains a fundamental question regarding how specific and multifaceted the link is between individual differences in intelligence and longevity. Evidence for an association with several leading causes of death has either not been replicated (dementia and respiratory disease),^7^ ^23^ is conflicting (different cancer sites),^5^ ^7^ ^8^ ^24^ ^25^ ^26^ ^27^ or is hitherto untested (digestive system disease). At least six publications have compared associations between premorbid intelligence and a selection of cause specific mortalities,^5^ ^7^ ^8^ ^10^ ^24^ ^28^ including some well characterised and extremely large cohorts with extensive follow-up; however, over-representation of men only samples^7^ ^10^ and follow-up that was terminated in middle age^5^ ^10^ has limited the generalisability of findings. Furthermore, low numbers of events for diseases common in older adult populations could have contributed to some apparently conflicting results.^8^ ^24^ ^28^

We investigated the magnitudes of the association between childhood intelligence and all major causes of death, using a whole year of birth population followed up to older age, therefore capturing sufficient numbers of cases for each outcome. Secondly, we investigated sex differences in the associations. Thirdly, we carried out sensitivity analyses to test for some possible mechanisms of association, including confounding and mediation by socioeconomic status.

## Methods

### Study population and data sources

In this prospective cohort study, all individuals born in Scotland in 1936 and registered at school in Scotland in 1947 were targeted for tracing and subsequent data linkage to death certificates. This was carried out by the National Records of Scotland, with permission from the registrar general of Scotland, using the National Health Service (NHS) central register for members traceable in Scotland, and the MRIS Integrated Database and Administration System for those in England and Wales. The confidentiality advisory group of the health research authority gave support under section 251 of the NHS Act 2006 for linkage without consent of mortality records including cause of death with intelligence scores age 11 from the Scottish Mental Survey 1947 (SMS1947).^25^ ^29^ ^30^

### Childhood intelligence

On 4 June 1947, about 94% of the Scottish population born in 1936 who were registered as attending school in Scotland (75 252) completed a test of general intelligence in the SMS1947 (n=70 805).^25^ ^29^ ^31^ This involved administration of the Moray House test No 12, which has 71 items tapping verbal and non-verbal reasoning ability.^29^ The test was introduced by school teachers who read aloud instructions before the start of a 45 minute test period. Each participant was allocated a score out of the maximum of 76 (some items scored more than one point). A recent follow-up study showed that the test had good concurrent validity (correlation coefficient about 0.8) with a well standardised individually administered Stanford version of the Binet test of intelligence in 1947^25^ ^29^ and with performance on Raven’s progressive matrices—a widely used non-verbal ability test (correlation coefficient about 0.7).^32^ The test has high lifelong stability of individual differences,^33^ ^34^ and several studies have provided good evidence for its external validity.^28^ ^35^ ^36^

### Cause specific death outcomes

Causes of death were coded according to the ICD-6-10 (international classification of diseases, 6th to 10th revisions) codes (see table A in appendix for codes). NHS (England and Wales) recorded deaths linked to cause of death data were provided for dates up to and including 31 December 2015, and respective data for NHSCR (Scottish) recorded deaths were provided up to 30 June 2015. Subsequent cause of death updates provided by NHSCR, on a quarterly basis up to 31 December 2015, were provided without unique ID numbers for individuals, and we therefore applied a matching process to link these to SMS1947 data (appendix).

### Patient involvement

No cohort participants contributed to the development of the present research question or the outcome measures, nor were they involved in the design, recruitment, or conduct of the study. There is no intention to disseminate the results of this electronic data linkage study to its participants.

### Statistical analysis

The analytic sample includes 65 765 sample members who were traced for linkage to mortality records and who had a census date (that is, a last known GP registration date for those who emigrated or joined the armed forces) and an intelligence test score from 1947 (fig 1). They represent 92.3% of those who took part in the SMS1947 and 87.4% of Scottish schoolchildren born in 1936. Exclusions because of missing intelligence test scores affected 6.8% (5103) of the total 1936 Scotland birth cohort, which has previously been reported,^25^ ^31^ and 6.6% (4744) of those who were successfully linked and had a census date.

**Figure f1:**
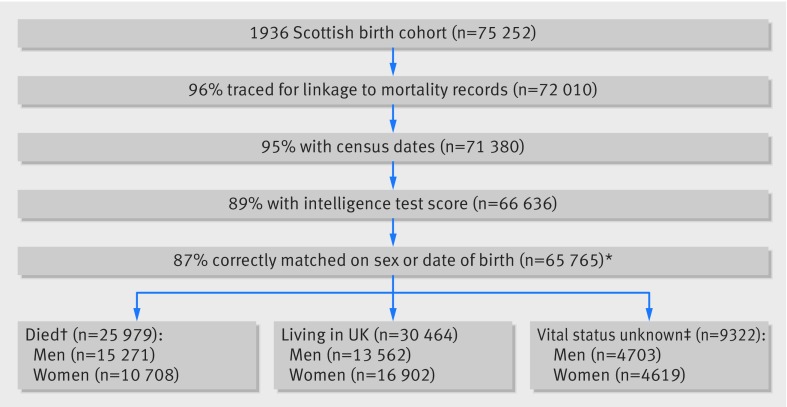
**Fig 1** Derivation of Scottish Mental Survey 1947 (SMS1947) analytic sample. Most of those with missing census dates had migrated or joined armed forces for whom records contained no last known GP registration date. *Analytic sample represents 92% of survey. †46.0% of those with vital status had died, which is approximate to Scottish population statistics for those aged 75-80. ‡Includes 5381 who emigrated abroad (2857 men); 3391 cancelled from GP registration (1383 men); 454 armed forces recruits (411 men); 96 residents of Northern Ireland or Isle of Man (52 men)

We conducted analyses with SPSS Statistics 21, unless otherwise stated. Cox proportional hazards regression models produced hazard ratios with 95% confidence intervals to summarise the relation between childhood intelligence and each cause of death, adjusted for age (in days) at cognitive testing and sex (dummy variable), for which there were no missing data. We ran models in which the cause of death was cardiovascular disease, coronary heart disease, stroke, cancer by type, respiratory disease, digestive disease, externally caused, injury, suicide, or dementia. Given that suicide is the likely cause of death in most death certificates that state “open verdict” or “undetermined intent,”^37^ we reported not only on models to predict deaths formally recorded as suicide but added deaths of undetermined intent to models of suicide. A cause of death was included if it was listed among multiple causes of death (MCOD) on the death certificate, but we repeated each model in which the endpoint was the underlying cause of death^38^ (see appendix).

In survival analyses, the time scale was calendar time (days) from the date of the intelligence test (4 June 1947) to the census date, which was the earliest of date of death; last known date of GP registration (for migration or armed forces entrants); or 31 December 2015 (end of follow-up). Members who died from a different cause to that being modelled were included in the denominator (people at risk) and censored at date of death, as is standard practice in epidemiological analyses. The proportional hazards assumption was assessed by inspection of log−log plots and formally tested in Stata 14 with the Schoenfeld residuals test. The assumption held for associations between intelligence and all major causes of death according to both methods, with the exception of deaths related to cancer with log−log plots and deaths from injury under formal testing (P=0.03). To visually inspect associations for linearity, we graphically plotted hazard ratios and their 95% confidence intervals to show the risk of each event type in accordance with intelligence test score in 10ths (for >1000 numbers of deaths) or quarters (for <1000 numbers of cases). For our main results, we estimated hazard ratios and their 95% confidence intervals for cause specific mortality according to a 1 SD (about 15 points) advantage in intelligence test score. With the suggestion that some associations between intelligence and death are modified by sex, we formally tested for sex differences by including an interaction term (sex × intelligence score) in models predicting cause of death. We then produced effect estimates separately for men and women. Finally, to adjust for multiple testing, using R,^39^ we made false discovery rate correction to significant P values resulting from all models^40^ and report on significant models (P<0.05) that failed this correction.

### Assessing selection bias caused by missing intelligence test data

We examined whether the 6.8% missing data on the intelligence test in the SMS1947 caused selection bias that affected the magnitude of the association between intelligence and mortality. We conducted sensitivity analyses using a representative subsample (1.7%; n=1208) of the SMS1947, the so called “6 day sample.”^25^ ^37^ ^38^ This subsample was affected by a similar degree of missing data as the whole SMS1947 on the Moray House test; however, they all took an additional, individually administered intelligence test on another occasion (see appendix for more details).

### Confounder adjustment: adjustment for school

In the absence of individual level data on socioeconomic status in the full SMS1947, we used school attended as a proxy measure to adjust for potential confounding by background socioeconomic status. A Scottish cohort study has shown that primary school has moderate correlation with paternal social class.^41^ Schools were specified as strata in the models for major causes of death and cancer subtypes, and fixed effects models adjusted for all characteristics shared by pupils from the same school were. As children could be selected into some schools because of previous cognitive ability, however, there might be over-adjustment in this particular sensitivity analysis.

### Confounder adjustment: subgroup analyses

We repeated models for leading causes of death in association with childhood intelligence scores in a representative subsample of the SMS1947, the so called “30 day sample” (7.2%; n=5083), on whom more background data were available to test for potential confounding (see appendix for more information). These data included socioeconomic status variables (paternal occupational status, home overcrowding, and school absenteeism) and indicators of physical status (height and physical disability).

### Adjustment for adult socioeconomic status and smoking: replication sample

In the absence of data in the intermediary “lifetime” period of the study with which to test for potential mediation, we report on equivalent models run on a replication sample—the “West of Scotland Twenty-07 study”—with and without adjustment for adult smoking status and occupational status. This cohort shares similarities to the SMS1947, including Scotland derived 1930s birth cohort and linkage to mortality records to at least 2015 (see appendix).

## Results

### Characteristics of the sample

Among 75 252 members of the 1936 birth cohort, 3242 were untraceable, 630 had no census dates (that is, migrated without a last known GP registration date), 4744 had no data on childhood intelligence, and 871 were mismatches of the linkage (fig 1). Among 65 765 individuals (32 229 were women; 5182 attended different schools) comprising the analytic sample, 25 979 had died and 30 464 were confirmed to be living in the UK at follow-up. Mean age at death was 66.1 (SD 10.6); mean time to follow-up was 57.0 (SD 18.4) years, and total person years of follow-up was 3.54 million. There were no marked differences in the characteristics of the analytic sample and the remainder of the SMS1947, except that, among those with a childhood mental test score, the analytic sample scored on average higher on the Moray House test (mean 37.1, SD 15.7) than those excluded from the analyses (35.4, SD 16.4; P<0.001).

### Childhood intelligence in relation to cause specific mortality

Figure 2 shows the associations between childhood intelligence (by 10ths or quarters) and risk of the major causes of death. These show that, for most endpoints, associations were inverse—that is, higher childhood intelligence was associated with a lower risk of cause specific death. Risk of death related to lifetime respiratory disease was two thirds lower in the top performing 10th for childhood intelligence versus the bottom 10th. Furthermore for deaths from coronary heart disease, stroke, smoking related cancers, digestive diseases, and external causes, risk of mortality was halved for those in the highest versus lowest 10th of intelligence. The risk of dementia related mortality and deaths by suicide were reduced by at least a third in the highest performing quarter of intelligence test score versus the lowest quarter. There was no evident association between childhood intelligence and mortality from cancers not related to smoking.

**Figure f2:**
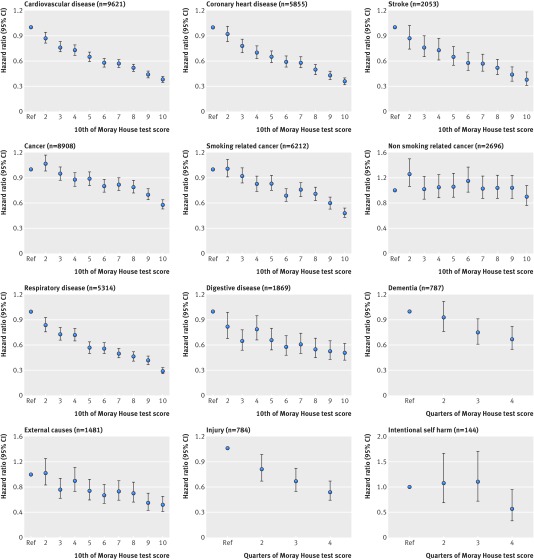
**Fig 2** Association between intelligence (Moray House test score) at age 11 and major causes of death (age and sex adjusted hazard ratios and 95% confidence intervals) to age 79 in Scottish Mental Survey 1947 (SMS1947)

Figure 3 shows the risk of major causes of death associated with a 1 SD higher score in childhood intelligence (see fig A in appendix for the equivalent plot where the outcome was underlying cause of death rather than MCOD). All hazard ratios showed inverse associations, and most showed narrow confidence intervals. The strongest effect sizes were seen for respiratory disease (hazard ratio 0.72, 95% confidence interval 0.70 to 0.74), coronary heart disease (0.75, 0.73 to 0.77), and stroke (0.76, 0.73 to 0.79). All others fell within the hazard ratio range 0.80-0.87, except for deaths from cancers not related to smoking cancer (0.96, 0.93 to 1.00)—the significance of this latter effect did not survive correction for false discovery rate. The relatively wide confidence interval for deaths from intentional self harm reflects the lower number of cases for this endpoint (0.87, 0.74 to 1.02). When deaths of undetermined intent were included in the model for suicide, the confidence interval fell below 1 (0.84, 0.74 to 0.96; 220 cases).

**Figure f3:**
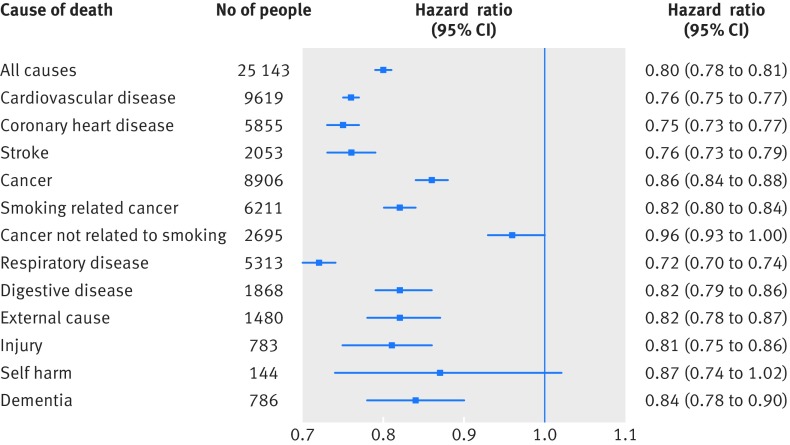
**Fig 3** Hazard ratios (95% confidence intervals) for association between 1 SD higher score in intelligence test score at age 11 and cause of death to age 79 in 65 765 people in Scottish Mental Survey 1947 (SMS1947). Effect sizes are adjusted for sex and age at cognitive testing

Sex interaction effects were computed with childhood intelligence in the total sample, and sex specific hazard ratios were also estimated for the associations between childhood intelligence and leading causes of death (table B in appendix). In models of the total sample, interaction terms for sex × intelligence were significant for risk of cardiovascular disease (P<0.001), coronary heart disease (P<0.001), smoking related cancers (P=0.02), respiratory diseases (P<0.001), and dementia (P=0.02). Though the inverse patterns of the association between intelligence and mortality observed for the total sample were seen in both men and women, the magnitude of the associations were slightly greater among women than among men for most cause specific deaths. The greatest difference in effect estimate was for dementia, whereby a 1 SD higher score in childhood intelligence was associated with a 10% reduced risk of death from dementia in men and a 24% reduced risk in women. Otherwise, between men and women there were equivalent hazard ratios for deaths from cancer not related to smoking and external causes of death, except for deaths by intentional self harm, which were associated with childhood intelligence in men (hazard ratio 0.80, 95% confidence interval 0.66 to 0.96) but not women (1.15, 0.82 to 1.60). This sex difference was also evident for suicide when we included open verdict deaths (0.76, 0.66 to 0.89, in men; 1.05, 0.83 to 1.34, in women; P=0.03 for sex × intelligence interaction effect in the total sample).

### Childhood intelligence in association with death by specific cancer type

Figure 4 shows the associations between groupings of childhood intelligence score (10ths or quarters) and deaths related to 15 specific cancers. About half of these showed inverse patterns of association with a degree of linearity, including cancers of the oesophagus, colon or rectum, stomach, liver, lung, kidney, bladder, and blood. The strongest association was evident for death related to lung cancer: the risk in the highest performing 10th of childhood intelligence was reduced by two thirds compared with the lowest performing 10th. Cancers showing negligible or irregular associations with childhood mental ability included mouth, pancreas, skin, ovaries, breast (women only), prostate (men only), and brain or central nervous system.

**Figure f4:**
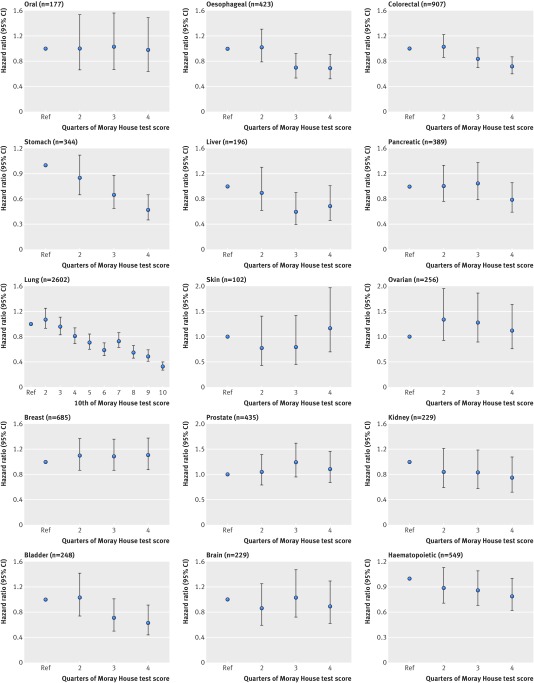
**Fig 4** Association between intelligence (Moray House test score) at age 11 and deaths from specific cancers (age and sex adjusted hazard ratios and 95% confidence intervals) to age 79 in Scottish Mental Survey 1947 (SMS1947)

Figure 5 shows the strengths of association between a 1 SD higher score in childhood intelligence and risk of death related to 15 specific cancers, with total cancers as a comparator (see fig B in appendix for equivalent forest plot reflecting underlying cause of death cases only). Whereas the effect estimate for death by any cancer clearly shows an inverse association with narrow confidence intervals (hazard ratio 0.86, 95% confidence interval 0.84 to 0.88), there is obvious heterogeneity across the results for specific cancer sites. Among the eight specific cancers that showed linearity in their inverse association with childhood mental ability in figure 4, the plot shows robust associations for seven of these (oesophageal, colorectal, stomach, liver, lung, bladder, and lymphoid or haematopoietic), with hazard ratios in the range 0.75-0.91. The strongest associations with childhood mental ability were deaths associated with lung cancer (0.75, 0.72 to 0.77) and stomach cancer (0.77, 0.69 to 0.85).

**Figure f5:**
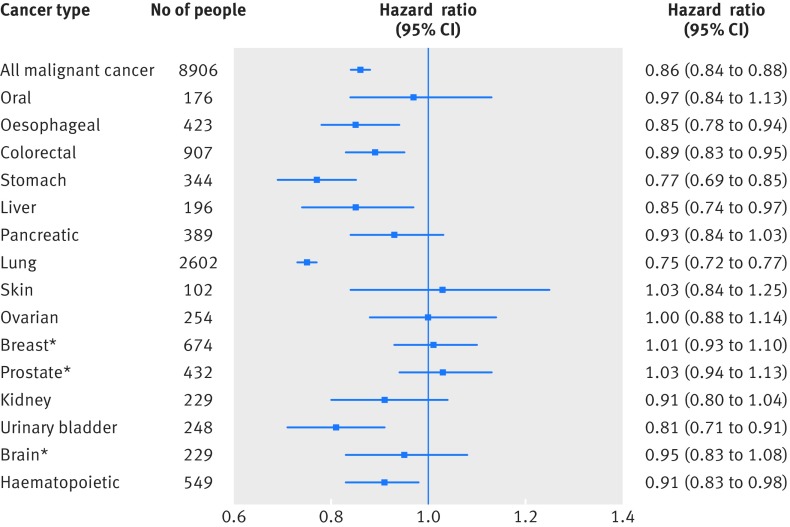
**Fig 5** Hazard ratios (95% confidence intervals) for association between 1 SD higher score in intelligence at age 11 and cause of death by cancer type to age 79 in Scottish Mental Survey 1947 (SMS1947). Effect sizes are adjusted for age at intelligence testing and sex, with exception of ovarian and breast cancer (women only) and prostate cancer (men only). *Non-smoking-related cancers (all others are smoking related)

We included an interaction term that included sex and childhood intelligence in models for IQ and specific cancer related mortality in the total sample and calculated sex specific hazard ratios (table C in appendix). These analyses showed no clear sex differences in direction or magnitude of the association between childhood intelligence and cancer specific mortality. The significant sex interaction effect (P=0.013) with mental ability in predicting lung cancer in the total sample could be a chance finding, and the direction of association was the same in men (hazard ratio 0.77, 95% confidence interval 0.74 to 0.81) and women (0.70, 0.66 to 0.75).

### Sensitivity analyses: assessment of selection bias caused by missing intelligence test data

In analyses of the so called “6 day sample” of the SMS1947 (that is, those born on the first day of the even numbered months of 1936), selection bias according to missing Moray House test scores in the SMS1947 did not affect the effect size of the association between intelligence test scores (on the Terman-Merrill, an individually administered test) and total mortality (see appendix).

### Sensitivity analyses: assessment of potential confounding

Firstly, we attempted to control for potential confounding by adjusting for the fixed effects of school attended in the full SMS1947. The hazard ratios for cause specific mortality and for deaths related to cancer subtype mostly showed a reduction by a small amount (attenuation range 0-30%) (see figs C and D in appendix), and the patterns of specificity were similar to those in the main analyses.

Secondly, in subgroup analysis of the SMS1947, including 2039 men and 1992 women in the 30 day sample, hazard ratios and their 95% confidence intervals showed inverse associations between childhood intelligence and all cause mortality and mortality related to cardiovascular disease, cancer (smoking related), respiratory disease, and digestive disease (but not cancers not related to smoking, external causes, and dementia, unlike in the total sample) (see table D in appendix). Respiratory disease held the strongest association with childhood intelligence (hazard ratio 0.73, 95% confidence interval 0.66 to 0.80), as in the full sample. In confounder adjusted models that included three indicators of childhood socioeconomic status, the relation between intelligence and all cause mortality or mortality from cardiovascular disease, any cancer, smoking related cancer, respiratory disease, or digestive disease was attenuated by 7-26%. Addition of physical status to the model had modest impact (attenuation range 10-26%).

### Sensitivity analyses: assessment of mediation

In a replication sample—the West of Scotland twenty-07 study—we observed similar hazard ratios to the SMS1947 for the associations between intelligence (tested in middle age) and specific underlying causes of death (fig C in appendix), though they were slightly stronger and with less precision. The one noticeable difference was for stroke as the underlying cause of death, which showed a weak association with midlife intelligence (hazard ratio 0.90, 95% confidence interval 0.70 to 1.15). After we adjusted for smoking, adult socioeconomic status, and self rated health, there were attenuation effects of 27-65% (table E in appendix), and associations remained the most robust for respiratory disease (0.77, 0.59 to 0.99) and coronary artery disease (0.79, 0.65 to 0.96).

## Discussion

In this first whole population birth cohort study linking childhood intelligence test scores to cause of death, in a follow-up spanning age 11-79, we found inverse associations for all major causes of death, including coronary heart disease, stroke, cancer, respiratory disease, digestive disease, external causes of death, and dementia. For specific cancer types the results were heterogeneous, with only smoking related cancers showing an association with childhood ability. In general, the effect sizes were similar for women and men (albeit marginally greater for women), with the exception of death by suicide, which had an inverse association with childhood ability in men but not women. In a representative subsample with additional background data, there was evidence that childhood socioeconomic status and physical status indicators had no more than a modest confounding impact on the observed associations. A replication study with adult data on smoking and socioeconomic status showed less than a quarter to two thirds percentage attenuation of the equivalent effect estimates for intelligence test performance in association with cause specific mortality.

### Strengths and weaknesses of this study

One weakness of the study is the absence of covariate data from childhood that could account for the effect estimates we observed in our main analysis. This lack of data, however, should be balanced with evidence from our sensitivity analyses, including a representative subsample of the cohort. Additionally, there are extensive reports from nearly all published studies that have attempted to control for potential confounding. In our own analysis of the whole SMS1947, adjustment for school led to little attenuation of the associations between intelligence and mortality. Because this is only a proxy for background socioeconomic status, and because it could lead to over-adjustment as some schools select on cognitive ability, however, we undertook further sensitivity analyses. In our own analysis of a representative subsample of the SMS1947, three indicators of background socioeconomic status, which are significant correlates of intelligence test scores, explained a quarter or less of the associations between intelligence and cause specific mortality. This is consistent with the main literature that has reported low attenuation effects in models of premorbid intelligence and all cause (such as in the meta-analysis by Calvin and colleagues^1^) or cause specific mortality, including cardiovascular disease,^4^ ^5^ ^10^ ^42^ ^43^ ^44^ cancer,^5^ ^26^ and external causes,^5^ ^10^ ^45^ including suicide^10^ ^15^ ^46^ and death from injury.^10^ ^16^ ^17^ Additional adjustments for perinatal factors and/or physical status indicators also show marginal (if any) attenuation effects,^4^ ^5^ as we observed here in our adjustment of height and physical disability. Other psychological factors that have been shown to correlate with intelligence test performance, including personality traits and mental health indicators, could yet partially explain these associations. In a previous paper on the 6 day sample of the SMS1947, however, we found that the personality trait of “dependability” (rated in adolescence and similar to the widely studied trait called conscientiousness), which was also predictive of mortality, did not account for the association with intelligence.^47^ In a different sample, the personality trait of neuroticism was shown to moderate, but not attenuate, the association between intelligence and death.^48^ The strength of some of the associations we reported, however, such as with respiratory disease, are perhaps too strong to be explained by any potential confounders we are aware of from the literature.

Another factor affecting validity of findings is historical inaccuracy of death certificates. Particular causes of death that could be under-reported by coroners include dementia,^49^ stroke,^50^ and suicide.^37^ Therefore, we judge that the greater chance of false negatives relative to false positives in the present study would act, if at all, to slightly attenuate the observed effect sizes. Indeed, a further analysis, in which we included “open verdict” deaths in the suicide model, increased the hazard ratio and narrowed its confidence interval.

Strengths of the present study include its analysis of a near complete year of birth cohort from a nation’s population, with a high return of linkage to all historical death records. The follow-up period from childhood through to older age enabled us to consider all major causes of death in the UK across the life course. Not only can we reproduce more accurately, and with low bias, results from previous cohorts on cardiovascular disease and external causes of death, but we have been able to make a substantial contribution to the literature on premorbid intelligence and risk of cancer and report for the first time on associations between childhood intelligence and death from diseases of the digestive system. Furthermore, our whole population sample of men and women enables the reporting of sex specific effects, which are often lacking from the literature, which draws heavily on data from male conscripts. For example, we have been able to report for the first time on the association between premorbid intelligence and the full range of subtypes of cancer in women. And we report for the first time on somewhat greater effect sizes among women relative to men for several causes of death, including respiratory disease and smoking related cancers.

### Comparison with other studies

An important aspect of the present study was to consider the role of premorbid intelligence in relation to specific cancer sites, given the considerable heterogeneity in cancer aetiology and conflicting findings from previous studies of an association between premorbid intelligence and risk of total cancer mortality.^5^ ^7^ ^8^ ^24^ ^25^ ^26^ ^27^ A cohort study of more than a million Swedish male conscripts—the largest to date—reported that, among 20 incident cancers, only three had significant associations with premorbid intelligence.^27^ The authors concluded that, with the likelihood of a few statistical false positives, cancer was unlikely to explain the association between premorbid intelligence and total mortality risk. Based on evidence from the present study, and other comparatively smaller cohorts, however, we would caution against over-generalising this summary. Our results are consistent with previously reported effect sizes for premorbid intelligence in association with lung cancer^5^ ^7^ ^8^ ^24^ and stomach cancer.^8^ ^24^ ^27^ Furthermore, we have shown, perhaps for the first time, that an association between previous intelligence and risk of cancer might be specific to types of smoking related cancer. We found significant inverse associations for five other smoking related cancers: oesophageal, colorectal, liver, bladder, and haematopoietic. Whereas two previous studies reported null findings for risk of colorectal and pancreatic cancer in association with childhood intelligence, there were low case numbers.^8^ ^24^ Indeed in a recent study of 728 165 Danish men, intelligence in early adulthood was significantly inversely associated with total gastrointestinal cancers.^7^ A pertinent question remains why more types of smoking related cancer were not related to premorbid intelligence in the study of a million Swedish men.^27^ This could be because of their much younger age at death ascertainment and/or national differences in prevalence of cigarette smoking. Sweden has been exemplary in its reduction in cigarette use (particularly among men) since the late 1960s,^51^ and maximum prevalence rates of smoking in Sweden at the time when the Swedish conscript study was conducted would have been much lower^52^ than equivalent rates in Scotland during our follow-up.^53^ If smoking is less prevalent in the Swedish population, and if smoking is an important mediator of the association between premorbid intelligence and risk of cancer, then this might explain the reduced and non-significant effect sizes reported by Batty and colleagues.^27^ One notable and significant finding from Sweden was a positive association between intelligence at conscription and incident skin cancer, indicating that higher ability was associated with an increased risk.^27^ Although the pattern of this relation in our own data corroborates this result, we did not observe a significant effect, perhaps because our older cohort grew up in a time before cheap flights to hot countries became ubiquitous and the proliferation in risk of skin cancer. The literature on premorbid intelligence and other cancer types is relatively small and/or underpowered. We report for the first time on the association with ovarian cancer in women, which we found to be null, and we replicate findings of previous studies of null associations with breast cancer in women^5^ ^8^ ^24^ and prostate cancer in men.^27^

Preliminary evidence of an inverse association between childhood intelligence and risk of dementia related death was reported from case-control studies of the 1932 Scottish Mental Survey that were linked to incident dementia records.^54^ ^55^ A recent prospective cohort study of this sample, followed up to 92 years, validated the finding.^23^ Our findings are consistent with this, as is evidence of a greater association with risk of dementia related mortality among women than men, also observed in the older Scottish cohort.^23^ This sex differential is also supported by evidence from individual participant meta-analyses showing that early school leaving in childhood is associated with death from dementia in women and not men.^56^

Mortality related to respiratory disease had the strongest association with premorbid intelligence in the present study (hazard ratio 0.72, 95% confidence interval 0.70 to 0.74). In a recent study of male conscripts in Denmark, which was the only previous study to report on the association, mortality from respiratory disease also had one of the strongest associations with premorbid intelligence (0.62, 0.60 to 0.65)^7^—only homicide was stronger in its association. Previous evidence that premorbid intelligence is associated with self reported chronic lung disease by midlife^57^ ^58^ and measured lung function in later life^59^ ^60^ ^61^ ^62^ supports these results. In contrast, a Scottish study reporting a weak and non-significant inverse association with incident respiratory disease might have been underpowered.^8^

Our own effect sizes for risk of mortality in association with premorbid intelligence corroborate those reported in the literature for all cause mortality (hazard ratio 0.80 versus aggregate hazard ratio of 0.80 in studies of ≥40 years’ duration),^1^ total cardiovascular disease (0.76 versus hazard ratios of 0.74-0.93),^7^ ^8^ ^9^ ^10^ ^63^ and coronary heart disease (0.75 versus hazard ratios of 0.70-0.86).^3^ ^4^ ^7^ ^9^ ^42^ ^43^ ^64^ The effect sizes for risk of stroke in association with premorbid intelligence have been less consistent in the literature, with significant inverse associations for incident or fatal only stroke observed in cohorts from Denmark, Scotland, Sweden, and the US^3^ ^4^ ^7^ ^65^ and negligible associations reported in separate cohorts from Denmark and Scotland.^8^ ^9^ ^42^ Weaker associations with stroke in previous studies are probably influenced by low numbers of stroke cases in cohort studies, driven in part by historical under-reporting of stroke in death certificates^50^ and/or relatively low numbers of ischaemic versus haemorrhagic strokes,^7^ particularly in cohort studies with follow-up in younger age groups. In our study cohort followed to older age, the observed effect size for risk of fatal stroke was equivalent in magnitude to that for coronary heart disease.

Our findings accord with previous reports of inverse associations between premorbid intelligence and risk of externally caused deaths, specifically from Danish and Scottish cohorts.^5^ ^7^ ^45^ In the Scottish study that followed up on deaths from ages 15 to 57, external causes of death held the strongest association with childhood intelligence relative to cancer, cardiovascular disease, and total mortality.^5^ In contrast, we found that external causes of death held the weaker association when compared with mortality from cardiovascular disease. It could be that differences in the composition of specific types of death that are externally caused in a younger versus older age cohort could influence the magnitude of this effect. Moreover, the hazard ratio we report for death from injury in association with childhood intelligence (0.81, 95% confidence interval 0.75 to 0.86) is similar in magnitude to that is a study of Danish men born in 1953, intelligence tested at age 12, and followed up to age 48 (0.82, 0.78 to 0.86).^16^ On the other hand, in studies where intelligence was tested in late childhood or early adulthood, the effect size in relation to death from injury has been greater (0.71 and 0.76, respectively).^7^ ^16^ ^17^ Several high powered prospective studies of cohorts of male conscripts have reported inverse associations between premorbid intelligence and later risk of suicide, including studies from Australia,^11^ Denmark,^7^ ^12^ and Sweden.^10^ ^13^ ^14^ ^15^ Far less is reported on the association in women and with follow-up to older ages. Our findings, however, are similar to the only previous study we are aware of to report on sex differentials for incident suicide risk in association with premorbid intelligence in a 40 year follow-up study, where an association was evident in men (age adjusted odds ratio of 0.90, 95% confidence interval 0.83 to 0.99) but negligible in women (1.04, 0.90 to 1.20).^66^

### Explanations and implications for future research

The strongest associations we observed were for natural causes of death related to coronary heart disease, stroke, respiratory disease, lung cancer, and stomach cancer. Smoking is a modifiable risk factor in each of these diseases, and higher premorbid intelligence has been related to lower likelihood of current smoking^67^ or past regular smoking^68^ and the increased likelihood of quitting smoking.^68^ ^69^ Indeed, in a recent comparison of effect sizes for intelligence and cause specific mortalities in Danish men, the authors made a similar summary of their own findings by implicating smoking as an important mediator in the context of natural causes of death.^7^ Nevertheless, when tested, smoking is no more than a partial mediator in associations between premorbid intelligence and total mortality,^3^ mortality related to coronary heart disease,^63^ and incident coronary heart disease or stroke.^64^ In our replication study, the addition of adult smoking showed small attenuating effects (12-27%) on the associations between midlife performance on intelligence tests and most cause specific mortalities, including coronary artery disease and respiratory disease, and had moderate attenuation effects on the associations with smoking related cancers and lung cancer (40% and 50%, respectively). Educational attainment or indicators of occupational status in adulthood have generally shown stronger attenuating effects.^1^ Though education is likely to fall on the causal pathway, after intelligence and before death, given evidence that differences in intelligence predict later outcomes of national school examinations,^70^ education might alternatively be a proxy indicator of intelligence and yet have no direct causal association with longer life. This is an ongoing discussion about possible over-adjustment by education in cognitive epidemiology.^71^ It is likely that the influence of a health behaviour (or pattern of behaviours) in explaining associations between premorbid function and cause specific mortality will be more precisely measured by future studies with repeat assessment across the life course, capturing cumulative risk. Indeed, follow-up of the Whitehall II study showed that four repeat measures of lifestyle behaviours accounted for far more of the socioeconomic-mortality gradient than baseline measurement alone,^72^ albeit this difference was evident for specific behaviours (especially physical activity) and not all (such as smoking). Alcohol consumption might have accounted for some of the association we observed between premorbid intelligence and digestive related mortality, and this could be similarly assessed. Alcohol related deaths were associated with premorbid intelligence in a 37 year follow up of the 1969-70 Swedish conscripts cohort.^73^ Childhood performance on intelligence tests was most strongly associated with deaths from respiratory disease. This might be attributable to a combination of prevalence of smoking and occupational related exposures. For many in this birth cohort, those in the working classes spent their early careers in mining and shipbuilding industries (men) or as cleaners and factory workers (women). Therefore, it is possible that, with the decline in rates of smoking and toxic exposures in the workplace, the magnitude of associations between IQ and death could be lower in more recent birth cohorts. Nevertheless, when we adjusted for background social class in our subgroup analyses, and for smoking and adult occupational status in our replication study, the risk of deaths from respiratory disease in relation to intelligence scores was only partially attenuated (by 12% and 33%, respectively).

Whereas the research literature has taken advantage of often rich lifestyle data to explore mediating effects on a pathway from premorbid intelligence to risk of mortality, recent evidence from longitudinal data from twins suggests the association might be largely caused by common genetic effects.^19^ With newly emerging data from genome-wide association studies (GWAS), there is likely to be a sizeable shift towards the testing of potential genetic factors, alongside environmental factors, in cognitive epidemiology. Recent GWAS data used in the context of the large UK Biobank sample has provided evidence for pleiotropy between midlife (premorbid) cognitive performance and genetic variants of specific diseases, including coronary heart disease, ischaemic stroke, and Alzheimers’ disease.^74^ Whether this is evidence for biological pleiotropy (thus supporting bodily system integrity theory) or a causal pathway from genetic variant to disease outcome, mediated by cognitive ability and subsequent health risk behaviours and/or occupational hazards, is the subject of ongoing work with Mendelian randomisation methods.^75^ There is the additional prospect of interaction between health risk behaviours and genetic markers for disease in explaining health differentials attributed to variation in intelligence, and none of these possibilities is mutually exclusive.

Because of the widely different causes of death in our study, there is no assumption of a “one size fits all” explanation of the observed effects. The association between premorbid intelligence and specific external causes of death warrants in depth and separate coverage. The specific association between premorbid intelligence and risk of suicide in men, for example, might need to be included in risk models alongside other psychosocial factors, such as the modifying effects of psychosis^66^ and high achieving parents,^46^ and the potentially confounding and mediating effects of depression and anxiety disorders^76^ and antisocial personality.^13^ ^15^ Furthermore, risk of suicide might be associated with premorbid intelligence through reduced opportunities in the job market, putting a greater strain on men than women in our society. Other cause specific mortalities, however, could be usefully studied together in their association with premorbid intelligence. For example, the underlying mechanisms in the association between premorbid intelligence and risk of mortality from dementia could have commonality with those of coronary heart disease and stroke. In support of this, previous evidence indicates that the effect on total dementia is driven by associations with vascular dementia and not Alzheimer’s disease.^54^ Furthermore, that risk of dementia mortality relates to premorbid intelligence for late onset but not early onset cases^55^—which tend to have a higher genetic component—might implicate health behaviours and lifestyle factors.

### Conclusions

We have been able to report on the strength of association between premorbid intelligence and a range of specific causes of death in a full population and with follow-up from childhood to near the end of the life course. The specialty of cognitive epidemiology remains in a growth period; therefore it is premature to make recommendations to practitioners and policymakers, though we have made a start on that elsewhere.^18^ Although we report that smoking and socioeconomic status are unlikely to fully mediate the observed associations, future studies would benefit from measures of the cumulative load of such risk factors over the life course. Finally, we highlight to researchers active in this specialty our robust findings of the association between premorbid intelligence and lifetime risk of mortality from coronary heart disease, stroke, smoking related cancers, respiratory disease, digestive related disease, external causes, and dementia, specifically the relatively strong lifetime associations with smoking related diseases. We also emphasise the marginally greater associated risks among women for coronary heart disease, smoking related cancers, respiratory disease, and dementia, which might indicate sex differential effects of intelligence on modifiable risk factors of disease.

**What is already known on this topic**

Prospective cohort studies has shown that higher intelligence tested in childhood or early adulthood is related to greater longevity
This relation is replicated for deaths related to cardiovascular disease related and external causes, though evidence for the association with cancer related mortality is conflicting, and associations with other leading causes of death (such as respiratory and digestive diseases) are largely unknown.
Previous studies have mostly been in male conscripts followed up to middle adulthood

**What this study adds**

This study reported on the association between childhood intelligence in all men and women born in one year in one country and specific causes of death in older adulthood
Intelligence tested in childhood was inversely associated with leading lifetime causes of death, including coronary heart disease, stroke, cancer, external causes, respiratory disease, digestive related diseases, and dementia. The results for specific cancer sites were heterogeneous, and significant only for smoking related cancers
Lower intelligence in childhood was consistently associated with most leading causes of death in women, as well as in men as has previously been reported.

## Web Extra

Extra material supplied by the author

## Notes

We thank the NHS Central Register (NHSCR) for linkage to Scottish death records and the Health and Social Care Information Centre (HSCIC) for linkage to Office of National Statistics (ONS) death records from England and Wales. We are also indebted to Alisdair Tullo from University of Edinburgh for custom building a program to match mortality cases without ID numbers to individuals in the SMS1947 and to Paul Redmond from the University of Edinburgh for receiving the linked data.

Contributors: CMC contributed to the design of the study, acquired the data, cleaned and analysed the data, interpreted the data, and drafted and revised the paper. GDB contributed to the design of the study, interpreted the data, and critically revised the paper. CEB managed the acquisition of data, interpreted the data, and critically revised the paper. AP and AT acquired the data and critically revised the paper. IC acquired the data, interpreted the data, and critically revised the paper. GD proposed and analysed the replication sample and critically revised the paper. IJD initiated the project, conceived and designed the study, interpreted the data, and critically revised the manuscript. CMC is guarantor.

Ethical approval: Not required.

Transparency: The lead author affirms that this manuscript is an honest, accurate, and transparent account of the study being reported; that no important aspects of the study have been omitted; and that any discrepancies from the study as planned (and, if relevant, registered) have been explained.

Data sharing: The full dataset is available for 10 years from the beginning of the study with permission of IJD at the University of Edinburgh

## References

[1] Calvin CM, Deary IJ, Fenton C, et al. Intelligence in youth and all-cause-mortality: systematic review with meta-analysis. Int J Epidemiol 2011;40:626-44. 10.1093/ije/dyq190 pmid:21037248. 21037248 PMC3147066

[2] Čukić I, Brett CE, Calvin CM, Batty GD, Deary IJ. Childhood IQ is associated with all-cause mortality in a full year-of-birth cohort: 68-year follow-up of 66 616 members of the Scottish Mental Survey 1947. Intelligence 2017;63:45-50. 28713184 10.1016/j.intell.2017.05.002 PMC5491698

[3] Batty GD, Wennerstad KM, Smith GD, et al. IQ in early adulthood and mortality by middle age: cohort study of 1 million Swedish men. Epidemiology 2009;20:100-9. 10.1097/EDE.0b013e31818ba076 pmid:19234402. 19234402

[4] Lawlor DA, Batty GD, Clark H, McIntyre S, Leon DA. Association of childhood intelligence with risk of coronary heart disease and stroke: findings from the Aberdeen Children of the 1950s cohort study. Eur J Epidemiol 2008;23:695-706. 10.1007/s10654-008-9281-z pmid:18704700. 18704700

[5] Leon DA, Lawlor DA, Clark H, Batty GD, Macintyre S. The association of childhood intelligence with mortality risk from adolescence to middle age: Findings from the Aberdeen Children of the 1950s cohort study. Intelligence 2009;37:520-8 10.1016/j.intell.2008.11.004.

[6] Batty GD, Shipley MJ, Mortensen LH, Gale CR, Deary IJ. IQ in late adolescence/early adulthood, risk factors in middle-age and later coronary heart disease mortality in men: the Vietnam Experience Study. Eur J Cardiovasc Prev Rehabil 2008;15:359-61. 10.1097/HJR.0b013e3282f738a6 pmid:18525394. 18525394

[7] Christensen GT, Mortensen EL, Christensen K, Osler M. Intelligence in young adulthood and cause-specific mortality in the Danish Conscription Database – A cohort study of 728,160 men. Intelligence 2016;59:64-71 10.1016/j.intell.2016.08.001.

[8] Hart CL, Taylor MD, Davey Smith G, et al. Childhood IQ, social class, deprivation, and their relationships with mortality and morbidity risk in later life: prospective observational study linking the Scottish Mental Survey 1932 and the Midspan studies. Psychosom Med 2003;65:877-83. 10.1097/01.PSY.0000088584.82822.86 pmid:14508035. 14508035

[9] Hart CL, Taylor MD, Smith GD, Whalley LJ, Starr JM, Hole DJ, et alChildhood IQ and cardiovascular disease in adulthood: Prospective observational study linking the Scottish Mental Survey 1932 and the Midspan studies. Soc Sci Med 2004;59(10 SPEC. ISS.):2131-8 10.1016/j.socscimed.2004.03.016. 15351478

[10] Hemmingsson T, Melin B, Allebeck P, Lundberg I. The association between cognitive ability measured at ages 18-20 and mortality during 30 years of follow-up--a prospective observational study among Swedish males born 1949-51. Int J Epidemiol 2006;35:665-70. 10.1093/ije/dyi321 pmid:16446349. 16446349

[11] O’Toole BI, Cantor C. Suicide risk factors among Australian Vietnam era draftees. Suicide Life Threat Behav 1995;25:475-88.pmid:8928202. 8928202

[12] Osler M, Nybo Andersen AM, Nordentoft M. Impaired childhood development and suicidal behaviour in a cohort of Danish men born in 1953. J Epidemiol Community Health 2008;62:23-8. 10.1136/jech.2006.053330 pmid:18079329. 18079329

[13] Allebeck P, Allgulander C, Fisher LD. Predictors of completed suicide in a cohort of 50,465 young men: role of personality and deviant behaviour. BMJ 1988;297:176-8. 10.1136/bmj.297.6642.176 pmid:3408955. 3408955 PMC1834219

[14] Batty GD, Whitley E, Deary IJ, Gale CR, Tynelius P, Rasmussen F. Psychosis alters association between IQ and future risk of attempted suicide: cohort study of 1,109,475 Swedish men. BMJ 2010;340:c2506 10.1136/bmj.c2506 pmid:20522657. 20522657 PMC2881197

[15] Sörberg A, Allebeck P, Melin B, Gunnell D, Hemmingsson T. Cognitive ability in early adulthood is associated with later suicide and suicide attempt: the role of risk factors over the life course. Psychol Med 2012;43:49-60. 10.1017/S0033291712001043 pmid:22617391. 22617391

[16] Osler M, Andersen AM, Laursen B, Lawlor DA. Cognitive function in childhood and early adulthood and injuries later in life: the Metropolit 1953 male birth cohort. Int J Epidemiol 2007;36:212-9. 10.1093/ije/dyl261 pmid:17175543. 17175543

[17] Batty GD, Gale CR, Tynelius P, Deary IJ, Rasmussen F. IQ in early adulthood, socioeconomic position, and unintentional injury mortality by middle age: a cohort study of more than 1 million Swedish men. Am J Epidemiol 2009;169:606-15. 10.1093/aje/kwn381 pmid:19147741. 19147741 PMC2640161

[18] Deary IJ, Weiss A, Batty GD. Intelligence and Personality as Predictors of Illness and Death: How Researchers in Differential Psychology and Chronic Disease Epidemiology Are Collaborating to Understand and Address Health Inequalities. Psychol Sci Public Interest 2010;11:53-79. 10.1177/1529100610387081 pmid:26168413. 26168413

[19] Arden R, Luciano M, Deary IJ, et al. The association between intelligence and lifespan is mostly genetic. Int J Epidemiol 2016;45:178-85. 10.1093/ije/dyv112 pmid:26213105. 26213105 PMC4795559

[20] Deary IJ. Looking for ‘system integrity’ in cognitive epidemiology. Gerontology 2012;58:545-53. 10.1159/000341157 pmid:22907506. 22907506

[21] Lubinski D, Humphreys LG. Some bodily and medical correlates of mathematical giftedness and commensurate levels of socioeconomic status. Intelligence 1992;16:99-115 10.1016/0160-2896(92)90027-O.

[22] Lubinski D. Cognitive epidemiology: With emphasis on untangling cognitive ability and socioeconomic status. Intelligence 2009;37:625-33 10.1016/j.intell.2009.09.001.

[23] Russ TC, Hannah J, Batty GD, Booth CC, Deary IJ, Starr JM. Childhood Cognitive Ability and Incident Dementia: The 1932 Scottish Mental Survey Cohort into their 10th Decade. Epidemiology 2017;28:361-4. 10.1097/EDE.0000000000000626 pmid:28151744. 28151744 PMC5381709

[24] Deary IJ, Whalley JMS, Starr JM. IQ at age 11 and longevity. In: Finch CE, Robine JM, Christen Y, eds. *Brain and Longevity: Perspectives in Longevity* Springer; 2003:153-64 10.1007/978-3-642-59356-7_10.

[25] Deary IJ, Lawrence J, Whalley JMS.*A lifetime of intelligence: follow-up studies of the Scottish Mental Surveys of 1932 and 1947.*American Psychological Association, 2009 10.1037/11857-000.

[26] Batty GD, Mortensen LH, Gale CR, Shipley MJ, Roberts BA, Deary IJ. IQ in late adolescence/early adulthood, risk factors in middle age, and later cancer mortality in men: the Vietnam Experience Study. Psychooncology 2009;18:1122-6. 10.1002/pon.1521 pmid:19189278. 19189278

[27] Batty GD, Wennerstad KM, Smith GD, et al. IQ in early adulthood and later cancer risk: cohort study of one million Swedish men. Ann Oncol 2007;18:21-8. 10.1093/annonc/mdl473 pmid:17220284. 17220284

[28] Deary IJ, Whiteman MC, Starr JM, Whalley LJ, Fox HC. The impact of childhood intelligence on later life: following up the Scottish mental surveys of 1932 and 1947. J Pers Soc Psychol 2004;86:130-47. 10.1037/0022-3514.86.1.130 pmid:14717632. 14717632

[29] Maxwell J. The level and trend of national intelligence: the contribution of the Scottish mental surveys (no. 48).University of London Press, 1961.

[30] Brett CE. Realising health data linkage from a researcher’s perspective: following up the 6-Day Sample of the Scottish Mental Survey 1947. Longit Life Course Stud 2014;5:283-98 10.14301/llcs.v5i3.266.

[31] Thomson GH. The trend of Scottish intelligence.University of London Press, 1949.

[32] Deary IJ, Whiteman MC, Starr JM, Whalley LJ, Fox HC. The impact of childhood intelligence on later life: following up the Scottish mental surveys of 1932 and 1947. J Pers Soc Psychol 2004;86:130-47. 10.1037/0022-3514.86.1.130 pmid:14717632. 14717632

[33] Deary IJ, Whalley LJ, Lemmon H, Crawford JR, Starr JM. The stability of individual differences in mental ability from childhood to old age: Follow-up of the 1932 Scottish mental survey. Intelligence 2000;28:49-55 10.1016/S0160-2896(99)00031-8.

[34] Gow AJ, Johnson W, Pattie A, et al. Stability and change in intelligence from age 11 to ages 70, 79, and 87: the Lothian Birth Cohorts of 1921 and 1936. Psychol Aging 2011;26:232-40. 10.1037/a0021072 pmid:20973608. 20973608

[35] Deary IJ, Gow AJ, Pattie A, Starr JM. Cohort profile: The lothian birth cohorts of 1921 and 1936. Int J Epidemiol 2012;41:1576-84. 10.1093/ije/dyr197 pmid:22253310. 22253310

[36] Deary ID, Whalley LJ, Starr JM. The Scottish Mental Surveys of 1932 and 1947.American Psychological Association, 2009 10.1037/11857-001.

[37] Gunnell D, Hawton K, Kapur N. Coroners’ verdicts and suicide statistics in England and Wales. BMJ 2011;343:d6030 http://www.ncbi.nlm.nih.gov/pubmed/21980064. 10.1136/bmj.d6030 pmid:21980064. 21980064

[38] Redelings MD, Sorvillo F, Simon P. A comparison of underlying cause and multiple causes of death: US vital statistics, 2000-2001. Epidemiology 2006;17:100-3. 10.1097/01.ede.0000187177.96138.c6 pmid:16357601. 16357601

[39] R Core Team. R: A language and environment for statistical computing.R Foundation for Statistical Computing, 2013.

[40] Benjamini Y, Hochberg Y. Controlling the False Discovery Rate : A Practical and Powerful Approach to Multiple Testing. JR Stat Soc 1995;57:289-300.

[41] Dundas R, Leyland AH, Macintyre S, Leon DA. Does the primary school attended influence self-reported health or its risk factors in later life? Aberdeen Children of the 1950s Study. Int J Epidemiol 2006;35:458-65. 10.1093/ije/dyi239 pmid:16284402. 16284402

[42] Batty GD, Mortensen EL, Nybo Andersen A-M, Osler M. Childhood intelligence in relation to adult coronary heart disease and stroke risk: evidence from a Danish birth cohort study. Paediatr Perinat Epidemiol 2005;19:452-9. 10.1111/j.1365-3016.2005.00671.x pmid:16269073. 16269073

[43] Kajantie E, Räikkönen K, Henriksson M, et al. Stroke is predicted by low visuospatial in relation to other intellectual abilities and coronary heart disease by low general intelligence. PLoS One 2012;7:e46841 10.1371/journal.pone.0046841 pmid:23144789. 23144789 PMC3492363

[44] Modig Wennerstad K, Silventoinen K, Tynelius P, Bergman L, Rasmussen F. Association between intelligence and type-specific stroke: a population-based cohort study of early fatal and non-fatal stroke in one million Swedish men. J Epidemiol Community Health 2010;64:908-12. 10.1136/jech.2008.084020 pmid:19833609. 19833609

[45] Meincke RH, Mortensen EL, Avlund K, Rosthøj S, Sørensen HJ, Osler M. Intelligence in early adulthood and mortality from natural and unnatural causes in middle-aged Danish men. J Epidemiol Community Health 2014;68:130-6. 10.1136/jech-2013-202637 pmid:24062410. 24062410

[46] Gunnell D, Magnusson PKE, Rasmussen F. Low intelligence test scores in 18 year old men and risk of suicide: cohort study. BMJ 2005;330:167 10.1136/bmj.38310.473565.8F pmid:15615767. 15615767 PMC544986

[47] Deary IJ, Batty GD, Pattie A, Gale CR. More intelligent, more dependable children live longer: a 55-year longitudinal study of a representative sample of the Scottish nation. Psychol Sci 2008;19:874-80. 10.1111/j.1467-9280.2008.02171.x pmid:18947352. 18947352

[48] Weiss A, Gale CR, Batty GD, Deary IJ. Emotionally stable, intelligent men live longer: the Vietnam Experience Study cohort. Psychosom Med 2009;71:385-94. 10.1097/PSY.0b013e318198de78 pmid:19251871. 19251871

[49] Thomas BM, Starr JM, Whalley LJ. Death certification in treated cases of presenile Alzheimer’s disease and vascular dementia in Scotland. Age Ageing 1997;26:401-6. 10.1093/ageing/26.5.401 pmid:9351485. 9351485

[50] Corwin LE, Wolf PA, Kannel WB, McNamara PM. Accuracy of death certification of stroke: the Framingham Study. Stroke 1982;13:818-21. 10.1161/01.STR.13.6.818 pmid:7147296. 7147296

[51] Wersäll JP, Eklund G. The decline of smoking among Swedish men. Int J Epidemiol 1998;27:20-6 10.1093/ije/27.1.20. 9563689

[52] Rodu B, Stegmayr B, Nasic S, Cole P, Asplund K. Evolving patterns of tobacco use in northern Sweden. J Intern Med 2003;253:660-5. 10.1046/j.1365-2796.2003.01143.x pmid:12755962. 12755962

[53] Taulbut M, Gordon D, McKenzie K. Tobacco smoking in Scotland: an epidemiology briefing. 2008. http://www.scotpho.org.uk/publications/reports-and-papers/493-tobacco-smoking-in-scotland-an-epidemiology-briefing-

[54] McGurn B, Deary IJ, Starr JM. Childhood cognitive ability and risk of late-onset Alzheimer and vascular dementia. Neurology 2008;71:1051-6. 10.1212/01.wnl.0000319692.20283.10 pmid:18579804. 18579804

[55] Whalley LJ, Starr JM, Athawes R, Hunter D, Pattie A, Deary IJ. Childhood mental ability and dementia. Neurology 2000;55:1455-9. 10.1212/WNL.55.10.1455 pmid:11094097. 11094097

[56] Russ TC, Stamatakis E, Hamer M, Starr JM, Kivimäki M, Batty GD. Socioeconomic status as a risk factor for dementia death: individual participant meta-analysis of 86 508 men and women from the UK. Br J Psychiatry 2013;203:10-7. 10.1192/bjp.bp.112.119479 pmid:23818534. 23818534 PMC3696876

[57] Der G, Batty GD, Deary IJ. The association between IQ in adolescence and a range of health outcomes at 40 in the 1979 US National Longitudinal Study of Youth. Intelligence 2009;37:573-80. 10.1016/j.intell.2008.12.002 pmid:19907663. 19907663 PMC2772900

[58] Wraw C, Deary IJ, Gale CR, Der G. Intelligence in youth and health at age 50. Intelligence 2015;53:23-32. 10.1016/j.intell.2015.08.001 pmid:26766880. 26766880 PMC4659286

[59] Deary IJ, Whalley LJ, Batty GD, Starr JM. Physical fitness and lifetime cognitive change. Neurology 2006;67:1195-200. 10.1212/01.wnl.0000238520.06958.6a pmid:17030752. 17030752

[60] Carroll D, Batty GD, Mortensen LH, Deary IJ, Phillips AC. Low cognitive ability in early adulthood is associated with reduced lung function in middle age: the Vietnam experience study. Thorax 2011;66:884-8. 10.1136/thoraxjnl-2011-200104 pmid:21709165. 21709165

[61] Richards M, Strachan D, Hardy R, Kuh D, Wadsworth M. Lung function and cognitive ability in a longitudinal birth cohort study. Psychosom Med 2005;67:602-8. 10.1097/01.psy.0000170337.51848.68 pmid:16046374. 16046374

[62] Vasilopoulos T, Kremen WS, Grant MD, et al. Individual differences in cognitive ability at age 20 predict pulmonary function 35 years later. J Epidemiol Community Health 2015;69:261-5. 10.1136/jech-2014-204143 pmid:25273357. 25273357 PMC4756634

[63] Batty GD, Shipley MJ, Gale CR, Mortensen LH, Deary IJ. Does IQ predict total and cardiovascular disease mortality as strongly as other risk factors? Comparison of effect estimates using the Vietnam Experience Study. Heart 2008;94:1541-4. 10.1136/hrt.2008.149567 pmid:18801778. 18801778 PMC2602751

[64] Hemmingsson T, v Essen J, Melin B, Allebeck P, Lundberg I. The association between cognitive ability measured at ages 18-20 and coronary heart disease in middle age among men: a prospective study using the Swedish 1969 conscription cohort. Soc Sci Med 2007;65:1410-9. 10.1016/j.socscimed.2007.05.006 pmid:17582667. 17582667

[65] Jokela M, Batty GD, Deary IJ, Silventoinen K, Kivimäki M. Sibling analysis of adolescent intelligence and chronic diseases in older adulthood. Ann Epidemiol 2011;21:489-96. 10.1016/j.annepidem.2011.01.008 pmid:21440456. 21440456

[66] Andersson L, Allebeck P, Gustafsson JE, Gunnell D. Association of IQ scores and school achievement with suicide in a 40-year follow-up of a Swedish cohort. Acta Psychiatr Scand 2008;118:99-105. 10.1111/j.1600-0447.2008.01171.x pmid:18331576. 18331576

[67] Batty GD, Deary IJ, Schoon I, Gale CR. Mental ability across childhood in relation to risk factors for premature mortality in adult life: the 1970 British Cohort Study. J Epidemiol Community Health 2007;61:997-1003. 10.1136/jech.2006.054494 pmid:17933959. 17933959 PMC2465619

[68] Batty GD, Deary IJ, Macintyre S. Childhood IQ in relation to risk factors for premature mortality in middle-aged persons: the Aberdeen Children of the 1950s study. J Epidemiol Community Health 2007;61:241-7. 10.1136/jech.2006.048215 pmid:17325403. 17325403 PMC2652919

[69] Taylor MD, Hart CL, Davey Smith G, et al. Childhood mental ability and smoking cessation in adulthood: prospective observational study linking the Scottish Mental Survey 1932 and the Midspan studies. J Epidemiol Community Health 2003;57:464-5. 10.1136/jech.57.6.464 pmid:12775797. 12775797 PMC1732467

[70] Deary IJ, Strand S, Smith P, Fernandes C. Intelligence and educational achievement. Intelligence 2007;35:13-21 10.1016/j.intell.2006.02.001.

[71] Deary IJ, Johnson W. Intelligence and education: causal perceptions drive analytic processes and therefore conclusions. Int J Epidemiol 2010;39:1362-9. 10.1093/ije/dyq072 pmid:20504860. 20504860

[72] Stringhini S, Sabia S, Shipley M, et al. Association of socioeconomic position with health behaviors and mortality. JAMA 2010;303:1159-66. 10.1001/jama.2010.297 pmid:20332401. 20332401 PMC2918905

[73] Sjölund S, Allebeck P, Hemmingsson T. Intelligence quotient (IQ) in adolescence and later risk of alcohol-related hospital admissions and deaths--37-year follow-up of Swedish conscripts. Addiction 2012;107:89-97. 10.1111/j.1360-0443.2011.03544.x pmid:21692890. 21692890

[74] Hagenaars SP, Harris SE, Davies G, et al. Shared genetic aetiology between cognitive functions and physical and mental health in UK Biobank (N=112 151) and 24 GWAS consortia. Mol Psychiatry 2015;2016:31120 pmid:26809841. 10.1038/mp.2015.225 PMC5078856 26809841

[75] Hagenaars SP, Gale CR, Deary IJ, Harris SE. Cognitive ability and physical health: a Mendelian randomization study. Sci Rep 2017;7:2651 10.1038/s41598-017-02837-3 pmid:28572633. 28572633 PMC5453939

[76] Gale CR, Deary IJ, Boyle SH, Barefoot J, Mortensen LH, Batty GD. Cognitive ability in early adulthood and risk of 5 specific psychiatric disorders in middle age: the Vietnam experience study. Arch Gen Psychiatry 2008;65:1410-8. 10.1001/archpsyc.65.12.1410 pmid:19047528. 19047528 PMC3634571

