# Supplementary material for: Childhood intelligence in relation to major causes of death in 68 year follow-up: prospective population study
Source: BMJ. 2017 Jun 28;357:j2708. doi: 10.1136/bmj.j2708 (PMC5485432; doi:10.1136/bmj.j2708)
Supplement: Supplementary file 1 — Appendix: Supplementary material [file calc037184.ww1.pdf]

Appendix: Supplementary Materials [posted as supplied by author]

Contents

Pages 2-4: Matching death records to individuals in Scottish Mental Survey 1947 database

Page 5: Assessing selection bias due to missing MHT scores using the 6-Day Sample, a representative subsample of the Scottish Mental Survey 1947

Page 6: Table A, on International Classification of Diseases for major causes of death

Pages 7-9: The 30-Day Sample: A representative subsample of the Scottish Mental Survey 1947

Pages 10-11: The West of Scotland Twenty-07 study: A replication sample

Page 12: Figure A, on associations between intelligence and underlying causes of death in the Scottish Mental Survey 1947

Page 13: Figure B, on associations between intelligence and cancer subtypes as underlying causes of death in the Scottish Mental Survey 1947

Page 14: Table B, on sex differences in associations between intelligence and major causes of death in the Scottish Mental Survey 1947

Page 15: Table C, on sex differences in associations between intelligence and death from specific cancers in the Scottish Mental Survey 1947

Page 16: Figure C, on associations between intelligence and causes of death in the Scottish Mental Survey 1947, adjusting for school

Page 17: Figure D, on associations between intelligence and cancer subtypes as causes of death in the Scottish Mental Survey 1947, adjusting for school

Page 18: Table D, on confounder-adjusted associations between intelligence and leading causes of death in the 30-Day subsample of the Scottish Mental Survey 1947

Page 19: Figure E, a cohort comparison of associations between intelligence and underlying causes of death: the Scottish Mental Survey 1947 and the West of Scotland Twenty-07 study

Page 20: Table E, on smoking and adult socioeconomic status-adjusted associations between intelligence and underlying causes of death in the West of Scotland Twenty-07 study

Matching death records to individuals in Scottish Mental Survey 1947

1. *Matching death records without ID numbers to individuals in the Scottish Mental Survey 1947 database*

Scotland-recorded deaths between 1 July 2015 and 31 December 2015 were provided to the study without unique ID numbers for individuals. Therefore an electronic matching program was run to amalgamate individuals' death data with their respective childhood data. The program used key variables (first name, surname, date of birth) to match individuals in the two datasets, utilising Python programming language (https://www.python.org/), the NumPy library for scientific computing (http://www.numpy.org/), and, the pandas library for data analysis (http://pandas.pydata.org/). In attempting to match 2453 individuals' death data with their childhood records, the program successfully matched 1939 individuals, whereas 514 failed, due largely to first name variants between the two files. Of the failed matches, 315 were resolved manually, leaving 199 non-matches that were excluded from analyses.

2. *Reconciling mismatches on sex and date of birth between individuals' records in the mortality death and the Scottish Mental Survey 1947 data*

Following the merging of files, there were numbers of mismatches on sex and date of birth identified between the historical records and the linkage data. There were 296 mismatches on sex and 3114 mismatches on date on birth. Manual checks on identifiable data were carried out to reconcile these differences (details below) resulting in 987 exclusions due to mismatching (178 of these also met other exclusion criteria).

Date of birth mismatches - There were 3114 mismatches on date of birth (4.3% of the traced sample), which is a similar proportion of mismatches to a previous linkage study in Scotland (Hart et al., Public Health, 2003). The table below summarises the differences in dates of birth between the two records. Firstly, we accepted mismatches as correct matches if there was a difference of 9 days or less in the dates, or, if

there was a difference of 10 days with only one digit different (n=1941). Secondly, we conducted manual checks on samples of mismatches, and confirmed a match if at least one of the following criteria were met: (1) where the name that matched was unusual; (2) where there was an unusual middle name that matched, (3) when there was a confirmed maiden name. This process of manual checking found correct matches for all mismatches where the different dates were exactly one month different, and, where day and month appeared in reverse to the other, and so we accepted these as true matches.

Reconciling differences in dates of birth between historical and linkage records

Difference in date of birth	N	Action	Result of manual checking	Final decision
1 day out	752	Accept	N/A	Accept
2 days out	330	Accept	N/A	Accept
3 days out	166	Accept	N/A	Accept
4 days out	115	Accept	N/A	Accept
5 days out	107	Accept	N/A	Accept
6 days out	74	Accept	N/A	Accept
7 days out	52	Accept	N/A	Accept
8 days out	53	Accept	N/A	Accept
9 days out	36	Accept	N/A	Accept
10 days out (1 digit out)	256	Accept	N/A	Accept
10 days out (2 digits out)	1	Check the 1 record	Uncertain	Exclude
11-30 days out	310	Check 20 records	80% correct; 20% uncertain	Exclude
1 month out	181	Check 20 records	95% correct; 5% uncertain	Accept
Different month	339	Check 20 records	53% correct; 47% uncertain	Exclude
Different month (1 digit out)	19	Check all 19 records	84% correct; 16% uncertain	Exclude
Different year	162	Check 20 records	75% correct; 15% uncertain	Exclude
2 digits out	1	Check all 3 records	1 correct; 2 uncertain	Exclude
Day and month in reverse	5	Check all 5 records	100% correct	Accept
Very different year	3	Check all 3 records	1 correct; 2 uncertain	Exclude
Different day and month	152	Check 20 records	60% correct; 40% uncertain	Exclude
**Total**	3114			

Sex mismatches - Participants' names were first checked to ensure compatibility between records, and then the forename helped to determine sex. Where there were name ambiguities on sex (n=9), AP, AT, CMC, and IJD, reached a consensus. One match was excluded from analyses due to a mismatch on both forename and date of birth.

Sensitivity analyses: Assessing selection bias due to missing MHT scores using the 6-Day Sample, a representative subsample of the Scottish Mental Survey 1947

We conducted sensitivity analyses on the 6-Day Sample (n=1208), a much smaller subsample, which is nevertheless representative of the Scottish Mental Survey 1947 (SMS1947) in terms of geographical spread and average test performance on the Moray House Test.[1] The 6-Day Sample were the 1936-born schoolchildren who were born on the 1st day of the even-numbered months. The 6-Day Sample completed, on a different day, a second, individually-administered intelligence test during the same year as the SMS1947 ("Terman-Merrill", which was a Stanford University revision of the Binet scale). The Terman-Merrill is highly correlated with scores on the Moray House Test, for those who had data on both (r=.80, n=1112). The 6-Day sub-sample had a rate of missingness on the SMS1947 of 8.0% (n=96), similar to that of the total sample (6.8%).

The hazard ratio (HR) and 95% confidence interval for all-cause mortality in association with a one SD higher Terman-Merrill score in the complete 6-Day Sample was 0.71 (95% CI = 0.65 to 0.78), where there were 490 deaths out of 1208 participants. Further, we estimated the HR for all-cause mortality in association with a one SD higher Terman-Merrill score for members of the 6-Day Sample for whom we also had data on the SMS1947 intelligence test (444 deaths out of 1112 participants), as: 0.71 (0.65 to 0.79). Finally, we estimated the equivalent HR for those 6-Day Sample members who had missing SMS1947 intelligence test data (46 deaths out of 96 participants), and the magnitude of the all-cause mortality risk estimate in association with a one SD higher Terman-Merrill score was also unchanged: 0.71 (0.54 to 0.93). This effect size is also similar (albeit slightly stronger) to that which we observed for the association between the Moray House intelligence test and all-cause mortality in the full SMS1947.

Members of the 6-Day Sample who were absentees of the SMS1947 (n=96) had on average a lower Terman-Merrill score (M=98.9, SD=23.7) compared to those who completed the SMS1947 (M=102.55, SD=20.09)—Cohen's *d* was 0.16. We conclude that an absence of lower ability scorers would have made little difference to our main results.

1. Macpherson JS. Eleven-Year-Olds Grow Up. London: University of London Press; 1958.

Table A

International Classification of Diseases, 6th, 7th, 8th, 9th and 10th Edition (ICD6, ICD7, ICD8, ICD9 and ICD10) codes for major causes of death

Primary Diagnosis	ICD-6 and ICD-7 Codes	ICD-8 Codes	ICD-9 Codes	ICD-10 Codes
Cardiovascular Disease	330-334, 410-456	393-448	390-448	I01-I78, G45
Coronary Heart Disease	420	410-414	402, 410-414, 429.2	I11, I20--I25
Cerebrovascular Disease (Stroke)	330-334	430-438	430-434, 436-438	I60-I69, G45, G46.3-.7
All malignant cancers	140-209	140-209	140-172, 174-209	C00-C43, C45-C97
Lip, oral cavity and pharynx	140-148	140-149	140-149	C01, C12, C00-C14
Oesophagus	150	150	150	C15
Colorectal	153-154	153-154	153-154	C18-C21
Stomach	151	151	151	C16
Liver and bile duct	155-156	155-156	155	C22
Pancreas	157	157	157	C25
Lung, bronchus	162-163	162	162	C33-C34
Skin	190-191	172-173	172-173	C43-C44
Ovarian (females only)	175	183	183	C56
Breast (females only)	170	174	174-175	C50
Prostate (males only)	177	185	185	C61
Kidney, renal pelvis and ureter	180	189	189	C64-C66
Urinary bladder	181	188	188-188	C67
Brain and central nervous system	193	191-192	191-192	C71
Lymphoid, haematopoietic and related tissue	200-205	200-207	200, 204-208	C81-C96
All smoking-related cancers	140-165, 171, 175, 180-181, 201,204	140-151, 153-155, 157, 160-162, 180, 183, 188, 189, 200, 204, 208	140-151, 153-155, 157, 160-162, 180, 183, 189, 188, 200, 204-208	C00-C16, C18-C22, C25, C30-C32, C34, C53, C56, C64-C67, C81-C86, C91-C95
All non-smoking-related cancers	Remaining malignant cancer codes after deducting smoking cancer codes	Remaining malignant cancer codes after deducting smoking cancer codes	Remaining malignant cancer codes after deducting smoking cancer codes	Remaining malignant cancer codes after deducting smoking cancer codes
Respiratory Diseases	470-527	460-519	460-519	J00-J99
Digestive System Diseases	530-587	520-577	520-579	K20-K93
External Causes of Death	E800-E999	E800-E999	E800-E999	V01-Y99
All accidents	E800-E936	E800-E929	E800-E929	V01-X59
Intentional self-harm (suicide)	E970-E979	E950-E959	E950-E959	X60-X84, Y87
Open verdict	-	E980-E989	E980-E989	Y10-Y34, Y87.2, Y89.9
Dementia	304-306	290, 293	290, 294, 331	F00-F01, F03, F09, G30, G31

The 30-Day Sample: A representative subsample of the Scottish Mental Survey 1947

Participants

The 36-Day Sample (N=7380; 10.4%) members of the Scottish Mental Survey 1947 (SMS1947) were those children born on the first, second, and third days of every month in 1936, who were selected for more thorough investigation in 1947, and who were similar to the full SMS1947 in terms of geographical representativeness and mean Moray House Test performance.[1] After excluding members of the 6-Day Sample – those born on the first day of even numbered months – who took part in annual follow-up visits into adulthood[2], we have selected the remainder (30-Day Sample) for the present sensitivity analyses, which have been the subject of previous investigations.[3,4] Just as for the full SMS1947, the linkage of members of the 30-Day Sample to their mortality records was given approval by National Services Scotland NHS Privacy Advisory Committee and the Confidentiality Advisory Group of the Health Research Authority.[5]

Measures

Intelligence, socioeconomic status, and physical status

The Moray House Test No. 12 was the measurement of intelligence, described in detail in the main manuscript. Covariate data that were recorded in 1947 around the same time as the intelligence test[6] included three variables indicating socioeconomic status, which can be used to account for potential confounding in the present study. These were: 1) *Father's, or head of household, occupation*, coded according to levels (from low to high status) using the 1951 Classification of Occupations[7]: unskilled jobs, semi-skilled, manual or non-manual skilled, intermediate, professional; 2) *Home occupancy*, calculated by dividing the number of individuals living in the child's home by the number of rooms within the home; and 3) *absent school days during the school year 1946-47*, calculated as a percentage. Confounding by physical status can be assessed in the present study by further controlling for height (inches), and the presence of physical disability (chorea, congenital paralysis, defective vision, deafness, encephalitis, epilepsy, and/or, meningitis).

Vital status

The procedures for tracing and linkage of the 30-Day Sample to death certificates are those detailed in the main manuscript for the SMS1947. Among 5083 of the historical sample with intelligence test scores, 4775 (93.9%) were traced for linkage to death records. By the follow-up date (31st December 2015), there were 1755 deceased, 2372 resident in Scotland, England or Wales, 357 embarked, and 23 Army conscripts.

Statistical analysis

The analytic sample included 4149 participants (81.6% of the 30-Day Sample) who were linked to mortality records, and who had complete data on intelligence test score, socioeconomic status and physical status measures. Missing intelligence test scores affected 452, or 9.1%, of the *30-Day Sample*, which is consistent with the full SMS1947 as detailed in the main manuscript. There were 1531 deaths recorded in the analytic sample

References

[1]Maxwell J. The level and trend of national intelligence: the contribution of the Scottish mental surveys (no. 48). London: University of London Press; 1961.

[2]Deary IJ, Whalley LW, Starr JM. A lifetime of intelligence: follow-up studies of the Scottish Mental Surveys of 1932 and 1947. Washington DC: American Psychological Association; 2009.

[3]Batty GD, Calvin CM, Brett CE, Čukić I, Deary IJ. Childhood body weight in relation to morbidity from cardiovascular disease and cancer in older adulthood: 67-year follow-up of participants in the 1947 Scottish Mental Survey. American journal of epidemiology. 2015 Nov 1;182(9):775-80.

[4]Batty GD, Calvin CM, Brett CE, Cukic I, Deary IJ. Childhood body weight in relation to cause-specific mortality: 67 year follow-up of participants in the 1947 Scottish Mental Survey. Medicine. 2016 Feb 1;95(6):e2263.

[5]Brett CE, Deary IJ. Realising health data linkage from a researcher's perspective: following up the 6-Day Sample of the Scottish Mental Survey 1947. Longitudinal and Life Course Studies. 2014 Oct 30;5(3):283-98.

[6]Scottish Council for Research in Education. The trend of Scottish intelligence: A comparison of the 1947 and 1932 surveys of the intelligence of eleven-year-old pupils. London: University of London Press; 1949.

[7]General Register Office. Classification of occupations 1951. London: Her Majesty's Stationary Office; 1956.

The West of Scotland Twenty-07 study: A replication sample

Participants

Participants are drawn from the West of Scotland Twenty-07 Study. This is a longitudinal study designed to investigate the social determinants of health and health inequalities. It comprises three narrow age cohorts who were aged 15, 35 and 56 at baseline in 1987/88. Study participants were randomly sampled from the Central Clydeside Conurbation, a mainly urban area centred on Glasgow city. They were interviewed in their homes by trained interviewers at baseline and on up to four further occasions over the following 20 years. Further details of the study are given elsewhere.[1] A comparison with data from the 1991 UK Census showed it to be representative of the underlying population in terms of sex, social class, car ownership and household tenure.[2] Data used in the replication study pertain to the oldest of the three cohorts obtained at baseline (N=1551) plus vital status obtained by record linkage. The birth years of the oldest cohort were: 1930 (n=60), 1931 (n=438), 1932 (n=825), and, 1933 (n=39).

Measures

Alice Heim 4 test of General Intelligence (AH4)

Part I of the AH4[3] was administered to the oldest cohort of the Twenty-07 study at baseline. The AH4 was designed for use with a cross section of the adult population and part I consists of 65 questions which measure verbal and numeric cognitive abilities. It was administered according to the instructions in the test manual. The result used here is the total number of questions answered correctly within the time limit of 10 minutes. For comparability this total score is standardised to zero mean and unit standard deviation (z-scored).

Vital Status

Vital status was ascertained via linkage to the UK's NHS central register. Participants of the Twenty-07 study were 'flagged' at the NHS central register at the outset of the study and since then regular notifications of deaths, embarkations and re-entries have been provided to the study. These notifications include date and cause of death. Primary and secondary causes are given in textual and coded forms, the latter being a mixture of ICD versions 9 and 10. The primary outcome here is the underlying cause of death grouped as shown in Supplementary Table 1. Of the analytic sample there were 820 deceased (men: 423) and 542 living by the census date (2nd February 2017).

Covariates

Smoking status was classified as current smoker/ex-smoker/never smoked. The Social Class measure is derived from the occupation of the head of household coded to the six fold Registrar General's classification: Professional/Managerial and technical/Skilled non-manual/Skilled manual/Semi-skilled/Unskilled.[4] Self-rated health (over the last twelve months) was recorded as excellent/good/fair/poor—this was dichotomised into excellent or good vs fair or poor. All covariates were treated as categorical when adjusted for.

References

[1]Benzeval M, Der G, Ellaway A, et al. Cohort Profile: West of Scotland Twenty-07 Study: Health in the Community. International Journal of Epidemiology. 2009;38(5):1215-1223.

[2]Der G. A comparison of the West of Scotland Twenty-07 study sample and the 1991 census SARs. Glasgow, 1998

[3]Heim AW. AH 4 Group Test of General Intelligence. Manual. By A (lice) W (inifred) Heim. Rev. Ed. NFER Publishing Company; 1970.

[4]OPCS. Classification of Occupations. HMSO. London, 1980.

Figure A

Hazard ratios (95% confidence intervals) for the association between a one SD higher score in intelligence test score at age 11 years and underlying cause of death in the Scottish Mental Survey 1947, N=65 765. Hazard ratios (HR) are adjusted for age at intelligence testing, and sex.

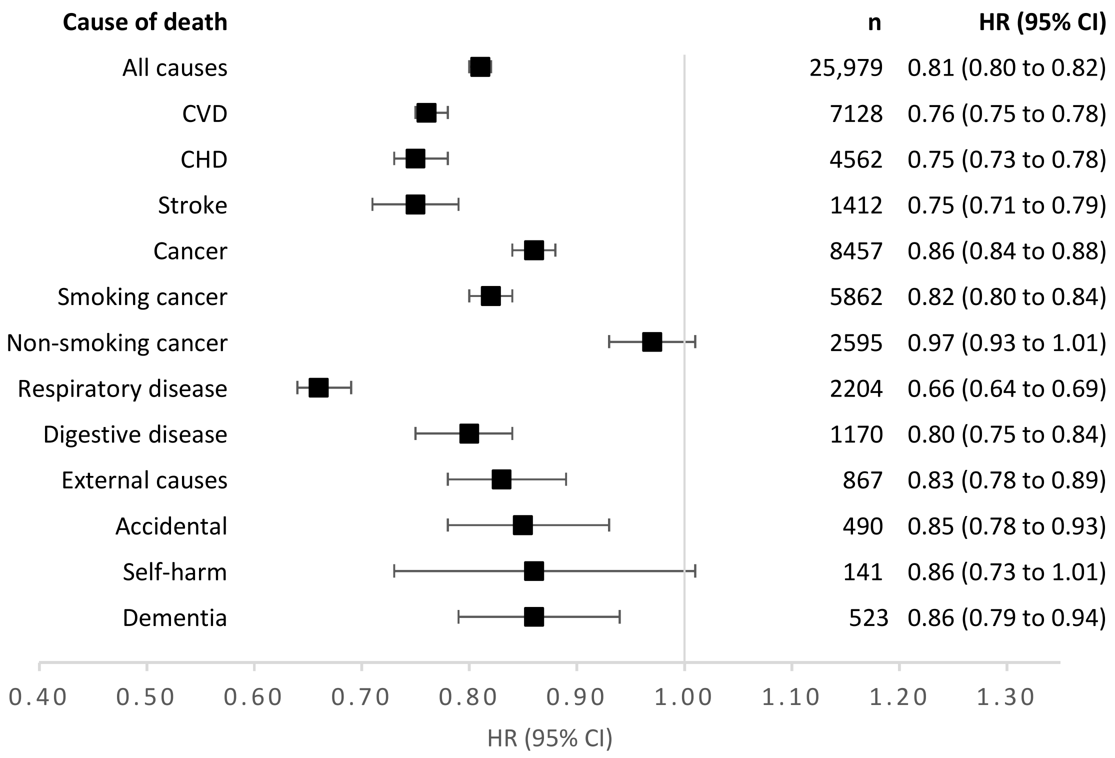

Figure B

Hazard ratios (95% confidence intervals) for the association between a one SD higher score in intelligence test score at age 11 years and underlying cause of death by cancer type to age 79 years in the Scottish Mental Survey 1947. Hazard ratios (HR) are adjusted for age at intelligence testing, and sex, with the exceptions of ovarian and breast cancer (women only), and, prostate cancer (men only).

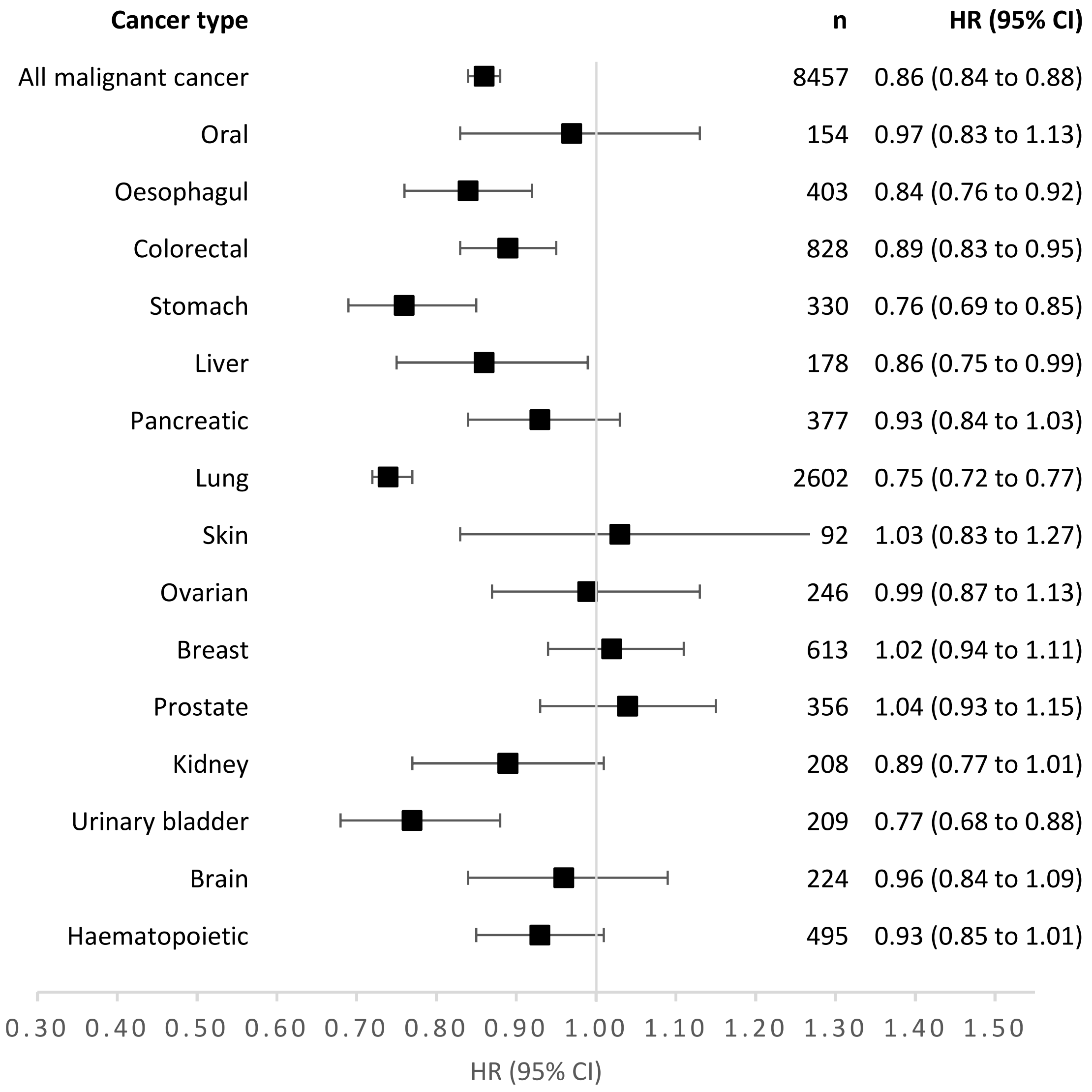

Table B

Hazard Ratios (and 95% confidence intervals) for a one SD higher score in intelligence score at age 11 years in relation to major causes of death in men and women separately in the Scottish Mental Survey 1947

Cause of death	Males		Females		p-value for sex by MHT interaction effect
	N	HR (95% CI)	N	HR (95% CI)	
All causes	15 271	0.83 (0.82 to 0.84)	10 708	0.78 (0.77 to 0.80)	<0.001
Cardiovascular disease	6070	0.79 (0.77 to 0.81)	3549	0.70 (0.68 to 0.73)	<0.001
Coronary heart disease	4045	0.78 (0.76 to 0.81)	1810	0.67 (0.64 to 0.70)	<0.001
Stroke	1114	0.78 (0.74 to 0.83)	939	0.72 (0.68 to 0.77)	0.07
Cancer	4890	0.87 (0.85 to 0.89)	4016	0.84 (0.82 to 0.87)	0.15
Smoking related	3614	0.84 (0.81 to 0.86)	2597	0.79 (0.76 to 0.82)	0.02
Non-smoking-related	1276	0.96 (0.91 to 1.02)	1419	0.96 (0.91 to 1.01)	0.87
Respiratory disease	3059	0.75 (0.73 to 0.78)	2254	0.68 (0.65 to 0.70)	<0.001
Digestive-system disease	1088	0.85 (0.80 to 0.90)	780	0.78 (0.73 to 0.84)	0.09
External cause	998	0.82 (0.78 to 0.87)	567	0.85 (0.78 to 0.93)	0.41
Accidental	505	0.82 (0.76 to 0.89)	278	0.77 (0.68 to 0.87)	0.54
Intentional self-harm	102	0.80 (0.66 to 0.96)	42	1.15 (0.82 to 1.60)	0.06
Dementia	414	0.90 (0.82 to 0.98)	373	0.76 (0.69 to 0.85)	0.02

N = 65 765. All HRs are adjusted for age at intelligence testing. MHT = Moray House Test.

Table C

Hazard Ratios (and 95% confidence intervals) for a one SD higher score in intelligence score at age 11 years with specific cancer-related deaths in men and women separately in the Scottish Mental Survey 1947

Cancer type	Males		Females		*p*-value for sex by MHT interaction effect
	N	HR (95% CI)	N	HR (95% CI)	
Oral	124	0.92 (0.78 to 1.09)	52	1.15 (0.85 to 1.56)	0.14
Oesophageal	293	0.87 (0.78 to 0.98)	130	0.81 (0.68 to 0.96)	0.72
Colorectal	558	0.92 (0.85 to 1.00)	349	0.83 (0.74 to 0.92)	0.09
Stomach	223	0.79 (0.69 to 0.89)	121	0.73 (0.61 to 0.88)	0.47
Liver	142	0.88 (0.75 to 1.04)	54	0.74 (0.57 to 0.98)	0.31
Pancreatic	203	0.98 (0.86 to 1.12)	186	0.87 (0.75 to 1.02)	0.36
Lung	1513	0.77 (0.74 to 0.81)	1089	0.70 (0.66 to 0.75)	0.01
Skin	58	1.05 (0.81 to 1.36)	44	0.99 (0.72 to 1.36)	0.73
Kidney	151	0.94 (0.81 to 1.10)	78	0.85 (0.67 to 1.06)	0.43
Bladder	162	0.82 (0.71 to 0.95)	86	0.77 (0.62 to 0.95)	0.53
Brain	136	0.92 (0.79 to 1.09)	93	1.00 (0.80 to 1.24)	0.67
Haematopoietic	341	0.87 (0.79 to 0.97)	208	0.98 (0.84 to 1.13)	0.18

N = 65 765. All HRs are adjusted for age at intelligence testing. Final column indicates *p*-values for interaction effects between sex and childhood intelligence in models that include the total sample. MHT = Moray House Test.

Figure C

Hazard ratios (95% confidence intervals) for the association between a one SD higher score in intelligence test score at age 11 years and cause of death in the Scottish Mental Survey 1947, adjusting for school (N=65 765). Hazard ratios (HR) are adjusted for age at intelligence testing, and sex.

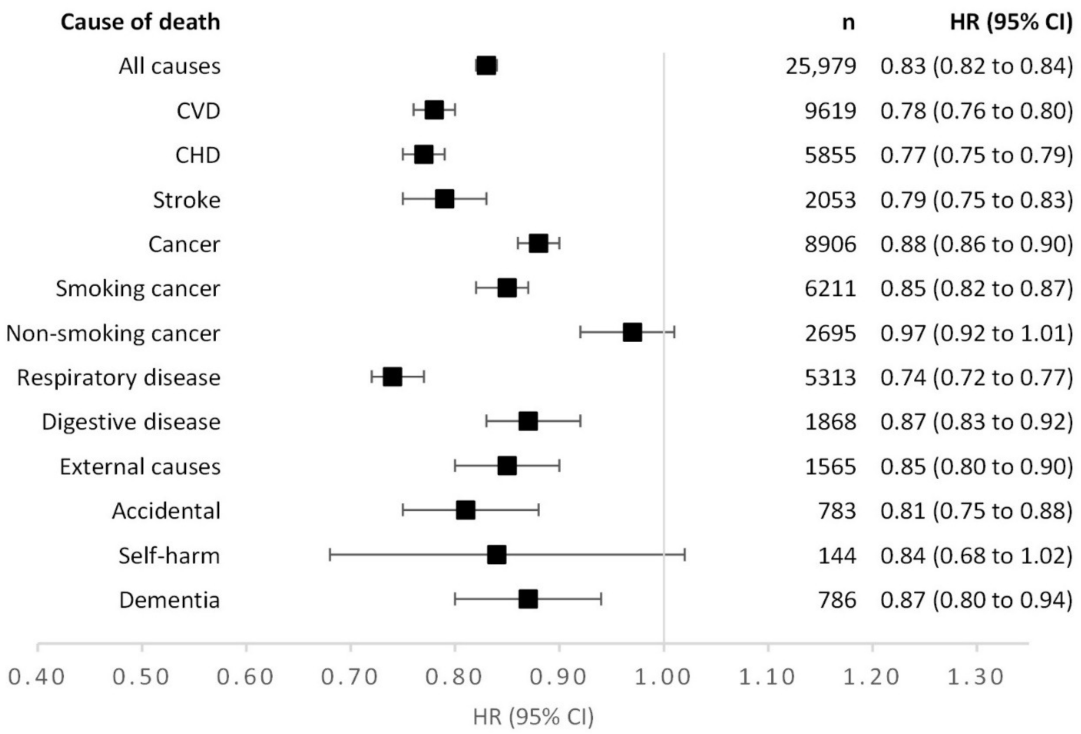

Figure D

Hazard ratios (95% confidence intervals) for the association between a one SD higher score in intelligence test score at age 11 years and cause of death by cancer type in the Scottish Mental Survey 1947, adjusting for school. Hazard ratios (HR) are also adjusted for age at intelligence testing, and sex, with the exceptions of ovarian and breast cancer (women only), and, prostate cancer (men only).

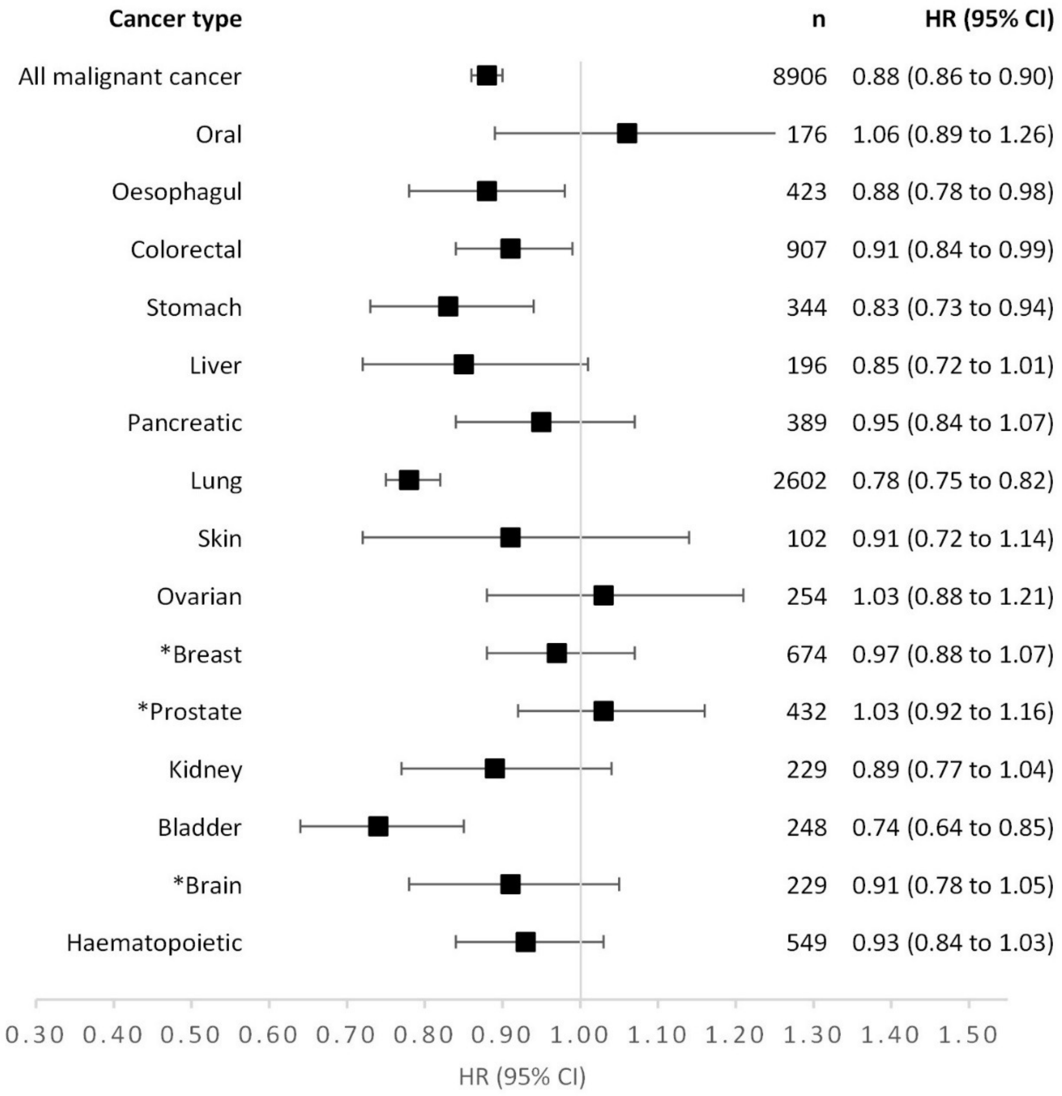

Table D

Hazard ratios (95% confidence intervals) for the association between a one SD higher intelligence test score at age 11 years and cause of death to age 79, accounting for childhood socioeconomic factors and physical status indicators in the 30-Day subsample[a] of the Scottish Mental Survey 1947

Cause of death	N cases	Model 1: Age, sex and school-adjusted	Model 2: Model 1 + background socioeconomic factors[b]	Model 3: Model 2 + physical status indicators[c]	% attenuation[d]: Model 1 to 2	% attenuation: Models 1 to 3
All causes	1531	0.82 (0.78 to 0.86)	0.84 (0.80 to 0.89)	0.85 (0.80 to 0.90)	15.8	18.9
Cardiovascular disease	700	0.80 (0.75 to 0.87)	0.82 (0.76 to 0.88)	0.82 (0.76 to 0.89)	7.1	10.1
Cancer	584	0.86 (0.79 to 0.94)	0.90 (0.82 to 0.98)	0.90 (0.82 to 0.98)	26.4	25.8
Smoking related cancer	407	0.83 (0.75 to 0.91)	0.86 (0.78 to 0.95)	0.86 (0.76 to 0.95)	21.3	20.6
Non-smoking cancer	177	0.95 (0.82 to 1.10)	0.98 (0.84 to 1.15)	0.98 (0.87 to 1.16)	70.1	69.4
Respiratory disease	367	0.73 (0.66 to 0.80)	0.76 (0.68 to 0.84)	0.75 (0.68 to 0.84)	12.7	11.5
Digestive-system disease	122	0.79 (0.67 to 0.95)	0.84 (0.70 to 1.01)	0.84 (0.70 to 1.01)	24.7	24.7
External cause	86	0.99 (0.80 to 1.23)	0.99 (0.79 to 1.24)	1.03 (0.82 to 1.30)	-	-
Dementia	52	1.02 (0.77 to 1.35)	1.00 (0.75 to 1.35)	1.03 (0.76 to 1.39)	77.2	32.5

[a]N = 4031, with complete data on all covariates (79.3% of 30-Day subsample).

[b]Childhood socioeconomic status indicators include: (1) father's or head of household's occupational status, (2) home overcrowding, (3) school absenteeism during 1946 to 1947

[c]Physical status indicators include: (1) height (cm) in 1947; (2) physical disability

[d]Percent attenuation = $100*((\beta_{\text{confounder-adjusted model}} - \beta_{\text{model 1}})/(\beta_{\text{model 1}}))$

Figure E

Hazard ratios (95% confidence intervals) for the association between a one SD higher score in premorbid intelligence test score and underlying cause of death by older adulthood, in the Scottish Mental Survey 1947 and West of Scotland Twenty-07 cohorts. Hazard ratios (HR) are adjusted for age at intelligence testing, and sex.

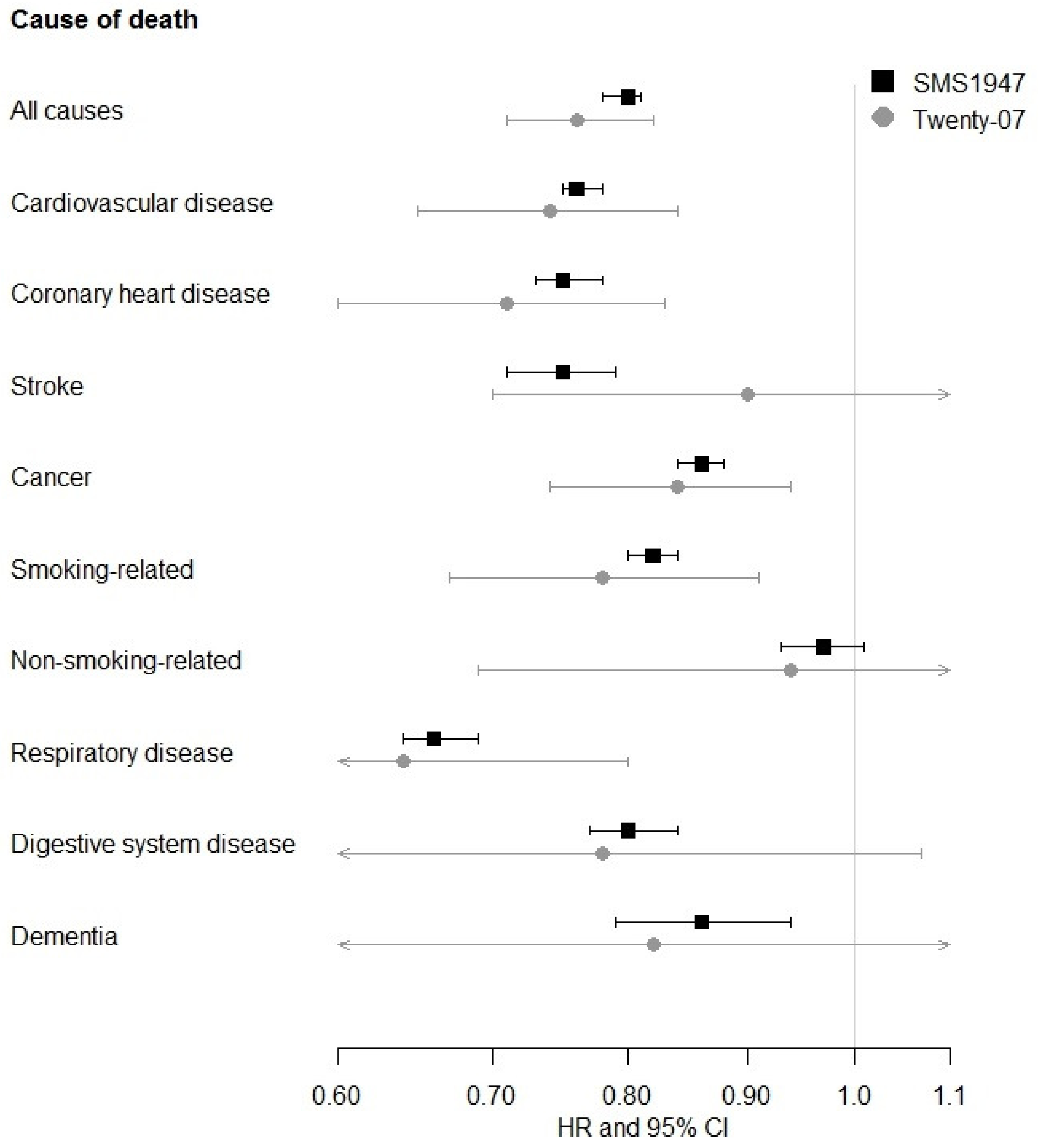

Table E

Hazard ratios (95% confidence intervals) for the association between a one SD higher Alice Heim 4 test score at age ~55 years and cause of death by older adulthood, accounting for smoking and socioeconomic status, in the West of Scotland Twenty-07 Study[a]

Cause of death	N cases	Model 1: Sex	Model 2: model 1 + smoking	Model 3: model 2 + socioeconomic status	Model 4: model 3 self-assessed health	% attenuation[b] Model 1 to 2	% attenuation Model 1 to 3	% attenuation Model 1 to 4
All causes	820	0.76 (0.71 to 0.82)	0.81 (0.75 to 0.87)	0.82 (0.75 to 0.89)	0.84 (0.77 to 0.91)	23.2	27.7	36.5
Cardiovascular disease	272	0.74 (0.65 to 0.84)	0.78 (0.68 to 0.89)	0.79 (0.68 to 0.91)	0.82 (0.70 to 0.95)	17.5	21.7	34.1
Coronary heart disease	163	0.71 (0.60 to 0.83)	0.74 (0.63 to 0.88)	0.75 (0.62 to 0.91)	0.79 (0.65 to 0.96)	12.1	16.0	31.2
Stroke	66	0.90 (0.70 to 1.15)	0.96 (0.74 to 1.24)	1.03 (0.77 to 1.37)	1.04 (0.78 to 1.39)	61.3	128.1	137.2
Cancer	290	0.84 (0.74 to 0.94)	0.88 (0.78 to 1.00)	0.87 (0.76 to 1.00)	0.88 (0.76 to 1.01)	26.7	20.1	26.7
Smoking related	188	0.78 (0.67 to 0.91)	0.86 (0.73 to 1.00)	0.86 (0.72 to 1.03)	0.87 (0.73 to 1.04)	39.3	39.3	44.0
Lung cancer	86	0.74 (0.59 to 0.92)	0.86 (0.68 to 1.10)	0.88 (0.68 to 1.14)	0.90 (0.69 to 1.18)	49.9	57.5	65.0
Non-smoking-related	42	0.94 (0.69 to 1.28)	0.87 (0.64 to 1.19)	0.81 (0.57 to 1.15)	0.80 (0.56 to 1.13)	-125.1	-240.6	-260.6
Respiratory disease	95	0.64 (0.51 to 0.80)	0.70 (0.55 to 0.88)	0.74 (0.58 to 0.95)	0.77 (0.59 to 0.99)	20.1	32.5	41.4
Digestive-system disease	41	0.78 (0.56 to 1.07)	0.80 (0.57 to 1.11)	0.76 (0.52 to 1.09)	0.75 (0.52 to 1.08)	10.2	-10.5	-15.8
Dementia	37	0.82 (0.58 to 1.15)	0.85 (0.60 to 1.20)	0.95 (0.65 to 1.39)	0.99 (0.68 to 1.46)	18.2	74.2	94.9

[a]N = 1362 (men = 613)
[b]Percent attenuation = $100*((\beta_{\text{confounder-adjusted model}} - \beta_{\text{model 1}})/(\beta_{\text{model 1}})$
